# Integrative Evidence Reveals Two New Species in the Millipede Genus *Coxobolellus* Pimvichai, Enghoff, Panha & Backeljau, 2020 and Reassigns *Benoitolus siamensis* (Attems, 1936) to the Genus *Pseudospirobolellus* Carl, 1912 (Diplopoda: Spirobolida: Pseudospirobolellidae)

**DOI:** 10.3390/ani16152304

**Published:** 2026-07-24

**Authors:** Piyatida Pimvichai, Brigitte Segers, Karin Breugelmans, Thierry Backeljau

**Affiliations:** 1 Department of Biology, Faculty of Science, Mahasarakham University, Maha Sarakham 44150, Thailand; 2 Royal Belgian Institute of Natural Sciences (RBINS), Vautierstraat 29, B-1000 Brussels, Belgium; 3 Evolutionary Ecology Group, University of Antwerp, Universiteitsplein 1, B-2610 Antwerp, Belgium

**Keywords:** identification key, mitochondrial DNA, monophyly, phylogeny, species delimitation

## Abstract

Two new millipede species of the genus *Coxobolellus* Pimvichai, Enghoff, Panha & Backeljau, 2020 (Diplopoda, Spirobolida: Pseudospirobolellidae) are described from southern Thailand based on morphological characters and mtDNA sequences (COI and 16S rRNA). The new species are: *Coxobolellus kaengkrachanensis* sp. nov. from Phetchaburi Province and *Coxobolellus planiprocessus* sp. nov. from Prachuap Khiri Khan Province. Both species are distinguished from all congeners by the posterior gonopod telopodite terminating apically in a coarsely serrate lamella. They differ from each other in the shape of the apical process of the posterior gonopod telopodite, being either a pointed spine in *Coxobolellus kaengkrachanensis* sp. nov. or a broad, flattened process in *Coxobolellus planiprocessus* sp. nov. The mean interspecific sequence divergence between the two new species is 0.08 for COI and 0.09 for 16S rRNA, while their mean interspecific sequence divergences with respect to other *Coxobolellus* species are 0.12 for COI and 0.15 for 16S rRNA. Phylogenetic analyses of COI and 16S rRNA firmly place the two new species as sister taxa within the *Coxobolellus* clade and reassign another pseudospirobolellid species, viz. *Benoitolus siamensis* (Attems, 1936), to the genus *Pseudospirobolellus*. Hence this latter species should be referred to as *Pseudospirobolellus siamensis* comb. nov. This conclusion is supported by a re-interpretation of the original description of the species’ gonopod morphology. In contrast, the phylogenetic analysis of COI sequences places *Benoitolus birgitae* (Hoffman, 1981) with strong support in the genus *Litostrophus* Chamberlin, 1921, within the family Pachybolidae, thus raising the question as to whether the genus *Benoitolus* can be maintained in the family Pseudospirobolellidae.

## Simple Summary

This study used both morphological characteristics and DNA evidence to improve the classification of millipede species in Thailand. Researchers discovered and described two new species, increasing the number of known species in the genus *Coxobolellus* to 15 and revealing that the group’s diversity is greater than previously recognized. The study also showed that *Benoitolus siamensis* had been assigned to the wrong genus. By re-examining its morphology and comparing DNA sequences, the researchers confirmed that it belongs to the genus *Pseudospirobolellus* rather than *Benoitolus*. This finding resolves a long-standing morphological misinterpretation and demonstrates that combining detailed morphological studies with molecular techniques is an effective way to identify species, clarify evolutionary relationships, and correct past taxonomic errors. These findings improve scientific knowledge of biodiversity in Thailand, provide a stronger foundation for future research, and ensure the accurate documentation of biological diversity.

## 1. Introduction

The Southeast Asian millipede family Pseudospirobolellidae (Diplopoda: Spirobolida) is a poorly known taxon that until recently was supposed to comprise only two genera, viz. *Benoitolus* Mauriès, 1980 (2–3 species) and *Pseudospirobolellus* Carl, 1912 (2 species), with in total four [1,2] or five [3] species, pending on whether or not the endemic Thai species *Cyclothyrophorus siamensis* Attems, 1936 is assigned to *Benoitolus* [3,4,5]. However, since 2020 interest in the family Pseudospirobolellidae was boosted by the description of the genera *Coxobolellus* Pimvichai, Enghoff, Panha & Backeljau, 2020 (with hitherto 13 species) and *Siliquobolellus* Pimvichai, Enghoff, Panha & Backeljau, 2022 (with hitherto 3 species), so that currently the family comprises four genera with 20–21 species [6,7,8,9], again depending on how *Cyclothyrophorus siamensis* is handled. Yet, current practice is to assign it to *Benoitolus* [6,10,11].

Mitochondrial DNA sequence data show that each of the genera *Pseudospirobolellus*, *Coxobolellus* and *Siliquobolellus* constitutes a well-supported clade and that jointly they form a well-supported, more inclusive Pseudospirobolellidae clade [8]. The mtDNA sequence data of the genus *Benoitolus*, however, hitherto involved only a single COI fragment (660 bp) of *B. birgitae* (Hoffman, 1981), which surprisingly does not cluster with the Pseudospirobolellidae, but turns up as a long branch within the pachybolid genus *Litostrophus* Chamberlin, 1921 [6,8,9]. Obviously, this raises questions about the taxonomic composition of the family Pseudospirobolellidae on the one hand, and the status and affinities of the genus *Benoitolus*, on the other [6,7,9].

Against this background, the present study contributes to the taxonomy of the Pseudospirobolellidae by integrating gonopod morphology with mitochondrial DNA sequence data (COI and 16S rRNA) to (1) investigate the phylogenetic relationships of *Cyclothyrophorus siamensis* Attems, 1936 and reassign it from the genus *Benoitolus* to the genus *Pseudospirobolellus*, thus renaming it as *Pseudospirobolellus siamensis* comb. nov., and (2) describe two new pseudospirobolellid species, *Coxobolellus kaengkrachanensis* sp. nov. and *Coxobolellus planiprocessus* sp. nov., from southern Thailand.

## 2. Material and Methods

Live specimens were hand-collected in November 2022 in southern Thailand and were partly preserved in 70% ethanol for morphological study and partly placed in a freezer at −20 °C for DNA analysis.

This research was conducted respecting the Animal Care and Use regulations (numbers U1-07304-2560 and IACUC-MSU-123-111/2025) of the National Research Council of Thailand and Mahasarakham University.

### 2.1. Morphology

Gonopods were photographed using a digital camera manipulated by Helicon Remote software (version 3.1.1.w; HeliconSoft, Kharkiv, Ukraine). Image stacking was performed with Zerene Stacker Pro software (version T2026-05-04-0750; Zerene Systems, Richland, WA, USA). For scanning electron microscopy (SEM), samples were air-dried directly from ethanol and sputter-coated with gold for 60 s using a Hitachi MC1000 sputter coater. SEM images were taken using a Hitachi TM4000Plus microscope (Hitachi High-Tech Corporation, Tokyo, Japan) at the Central Laboratory of Mahasarakham University.

Gonopod drawings were prepared by combining photographs and observations made through a stereomicroscope (Zeiss Stemi 305Trino, Carl Zeiss Suzhou Co., Ltd., Suzhou, China). Taxonomic identification and classification were based on Pimvichai et al. [6,7,9]. All specimens have been deposited in the collections of the Chulalongkorn University Museum of Zoology (CUMZ).

### 2.2. Terminology and Abbreviations Used for the Structure of Gonopods and Vulvae [6]

at = telopodite of the anterior gonopod, distinctly seen in posterior view;

atc = anterior gonopod telopodites forming a canopy-like structure; 

cx = coxal part of anterior gonopod;

oeg = opening of efferent groove;

op = operculum of vulvae;

pcx = coxal part of posterior gonopod;

pt = telopodital part of the posterior gonopod.

### 2.3. DNA Extraction, Amplification and Sequencing

Total genomic DNA was extracted from legs using the NucleoSpin Tissue kit (Macherey-Nagel, Düren, Germany) following the manufacturer’s instructions. PCR amplifications and sequencing of the standard mitochondrial COI DNA barcoding fragment [12] and 16S rRNA were done as described by Pimvichai et al. [6]. The COI and 16S rRNA fragments were amplified with the primers LCO-1490 and HCO-2198 [13] for COI, and 16Sar and 16Sbr [14] for 16S rRNA. The new nucleotide sequences have been deposited in GenBank under accession numbers PZ310494–PZ310497 for COI and PZ311613–PZ311616 for 16S rRNA. Sample data and voucher codes are provided in Table S1.

### 2.4. Alignment and Phylogenetic Analysis

The COI data involved 67 specimens, representing 18 genera and 51 nominal species of ingroup taxa (Table S1). Two species of the order Spirostreptida (Harpagophoridae), viz. *Anurostreptus barthelemyae* Demange, 1961 and *Thyropygus allevatus* (Karsch, 1881) were used as outgroup.

Forward and reverse sequences were assembled and examined for errors and ambiguities using CodonCode Aligner (ver. 4.0.4, CodonCode Corporation). All sequences were verified against reference sequences in GenBank using the Basic Local Alignment Search Tool (BLAST, see https://blast.ncbi.nlm.nih.gov/Blast.cgi?PROGRAM=blastn&PAGE_TYPE=BlastSearch&LINK_LOC=blasthome, accessed on 4 May 2026) provided by NCBI. Sequence alignment was performed with MUSCLE (ver. 3.6, see http://www.drive5.com/ muscle [15]), resulting in alignments of 660 bp for COI and 458 bp for 16S rRNA. Ambiguous nucleotide sites, saturation, and phylogenetic signal were assessed using DAMBE (ver. 5.2.65. see https://dambe.bio.uottawa.ca/DAMBE/dambe.aspx, accessed on 4 May 2026 [16]). MEGA (ver. 11.0.10, see http://www.megasoftware.net [17]) was employed to (1) screen sequences for stop codons, (2) translate nucleotide sequences into amino acids, and (3) calculate uncorrected pairwise p-distances by determining the proportion of different nucleotide sites between sequences.

Phylogenetic analyses included maximum likelihood (ML) and Bayesian inference (BI) methods. RAxML (ver. 8.2.12, see http://www.phylo.org/index.php/tools/raxmlhpc2_tgb.html, accessed on 4 May 2026; [18]) accessed through the CIPRES Science Gateway [19] was used to construct ML trees, applying a GTR + G (General Time Reversible + Gamma) substitution model with 1000 bootstrap replicates to evaluate branch support. BI trees were inferred using MrBayes (ver. 3.2.7a, see http://www.phylo.org/index.php/tools/mrbayes_xsede.html, accessed on 4 May 2026 [20]) accessed through the CIPRES Science Gateway [19]. Substitution models for the BI tree inference were selected on the basis of the Akaike Information Criterion scores in jModeltest (ver. 2.1.10, see https://www.github.com/ddarriba/jmodeltest2/releases, accessed on 4 May 202 [21]), identifying TrN + I + G (Tamura-Nei + Invariant + Gamma) (−lnL = 12,773.4615, gamma shape = 0.5230) as best model for COI, GTR + I + G (–lnL = 6620.4818, gamma shape = 1.0290 for 16S rRNA and GTR + I + G (General Time Reversible + Invariant + Gamma) (−lnL = 16,898.6693, gamma shape = 0.7900) for the combined COI + 16S rRNA dataset.

BI trees were run for 2 million generations with a heating parameter of 0.05 for COI, 0.07 for 16S rRNA and combined datasets, sampling every 1000 generations. Convergence was confirmed by ensuring that the standard deviations of split frequencies were below 0.01. The first 1000 trees were discarded as burn-in, and the final consensus tree was constructed from the remaining 3002 trees. Posterior probabilities were used to assess branch support.

For ML trees, branches were considered well-supported with bootstrap values (BV) of ≥70% [22], while values below 70% were regarded as indicating poor support. Similarly, branches in BI trees were considered well-supported with posterior probabilities (PP) of ≥0.95 [23], while values below this threshold were considered as indicating poor support.

### 2.5. Species Delimitation

Putative species were delimited using three species delimitation methods applied to the COI sequence data: Assemble Species by Automatic Partitioning (ASAP) (https://bioinfo.mnhn.fr/abi/public/asap/asapweb.html, accessed on 5 May 2026) [24], the General Mixed Yule Coalescent (GMYC) method [25], and the Poisson Tree Process (PTP) [26].

ASAP was run with K80 (=K2P) [27] distances using its default settings: split groups below 0.01 probability, keep 10 best scores, use fixed seed value −1, and highlight results between K80 distance values 0.005 and 0.05.

For GMYC an ultrametric tree was generated in BEAST (ver. 2.1.0, see http://tree.bio.ed.ac.uk/software/beast/, accessed on 5 May 2026 [28]) and an XML file was made with the BEAUti interface (ver. 2, see http://www.beast.community/) under a lognormal relaxed (uncorrelated) molecular clock with the GTR substitution model. Putative species were identified through single and multiple GMYC thresholds using the web interface at https://species.h-its.org/gmyc/ (accessed on 5 May 2026).

The PTP method was applied to a COI gene tree constructed with RAxML using https://species.h-its.org/ptp/ (accessed on 5 May 2026) to identify the most likely classification of branches into population and species-level processes [29].

## 3. Results

### 3.1. Phylogeny

The uncorrected p-distances between the COI sequences ranged from 0.00 to 0.26 (Table S2). The intraspecific COI sequence divergence of the two new species was 0.01 for *Coxobolellus kaengkrachanensis* sp. nov. and 0.02 for *Coxobolellus planiprocessus* sp. nov. The mean interspecific COI sequence divergence between the two new *Coxobolellus* species and the other *Coxobolellus* species was 0.12 ± 0.02 (range: 0.06–0.16), which was identical to the mean interspecific COI sequence divergence among the *Coxobolellus* species without the two new species (0.12 ± 0.02; range: 0.06–0.15). The mean interspecific COI sequence divergence between *C. kaengkrachanensis* sp. nov. and *C. planiprocessus* sp. nov. was 0.08 ± 0.00 (range: 0.08). The mean intergeneric COI sequence divergences between *Coxobolellus* and the other putative pseudospirobolellid genera were: 0.21 ± 0.01 (range: 0.20–0.23) for Pseudospirobolellus, and 0.17 ± 0.02 (range: 0.14–0.20) for *Siliquobolellus*. The mean intergeneric COI sequence divergence among *Coxobolellus*, *Pseudospirobolellus*, and *Siliquobolellus* was 0.19 ± 0.02 (range: 0.14–0.23).

The uncorrected p-distances between the 16S rRNA sequences ranged from 0.00 to 0.30 (Table S3). The intraspecific 16S rRNA sequence divergence of the two new species was 0.00 for *Coxobolellus kaengkrachanensis* sp. nov. and 0.01 for *Coxobolellus planiprocessus* sp. nov. The mean interspecific 16S rRNA sequence divergence between the two new *Coxobolellus* species and the other *Coxobolellus* species was 0.15 ± 0.02 (range: 0.09–0.19), while the mean interspecific 16S rRNA sequence divergence among the *Coxobolellus* species without the two new species was 0.11 ± 0.02 (range: 0.05–0.15). The mean interspecific 16S rRNA sequence divergence between *C. kaengkrachanensis* sp. nov. and *C. planiprocessus* sp. nov. was 0.09 ± 0.00 (range: 0.09–0.10). The mean intergeneric 16S rRNA sequence divergence between *Coxobolellus* and Pseudospirobolellus was 0.23 ± 0.03 (range: 0.18–0.28) (unfortunately there are no 16S rRNA sequence data for *Siliquobolellus*).

The uncorrected p-distances between the combined (COI+16S rRNA) sequences ranged from 0.00 to 0.27 (Table S4). The mean interspecific COI+16S rRNA sequence divergence between the two new *Coxobolellus* species and the other *Coxobolellus* species was 0.14 ± 0.02 (range: 0.08–0.17), while the mean interspecific COI+16S rRNA sequence divergence among the *Coxobolellus* species without the two new species was 0.11 ± 0.01 (range: 0.06–0.14). The mean interspecific COI+16S rRNA sequence divergence between *C. kaengkrachanensis* sp. nov. and *C. planiprocessus* sp. nov. was 0.09 ± 0.00 (range: 0.08–0.09). The mean intergeneric COI+16S rRNA sequence divergence between *Coxobolellus* and *Pseudospirobolellus* was 0.22 ± 0.01 (range: 0.19–0.24).

The sequence divergences between *Pseudospirobolellus avernus* and *P. siamensis* comb. nov. was 0.14 for COI, 0.09 for 16S rRNA, and 0.12 for COI+16S rRNA. The sequence divergences between *P. siamensis* comb. nov. and *Pseudospirobolellus* sp. KSM was 0.06 for COI, 0.09 for 16S rRNA, and 0.07 for COI+16S rRNA.

**Figure 1:**
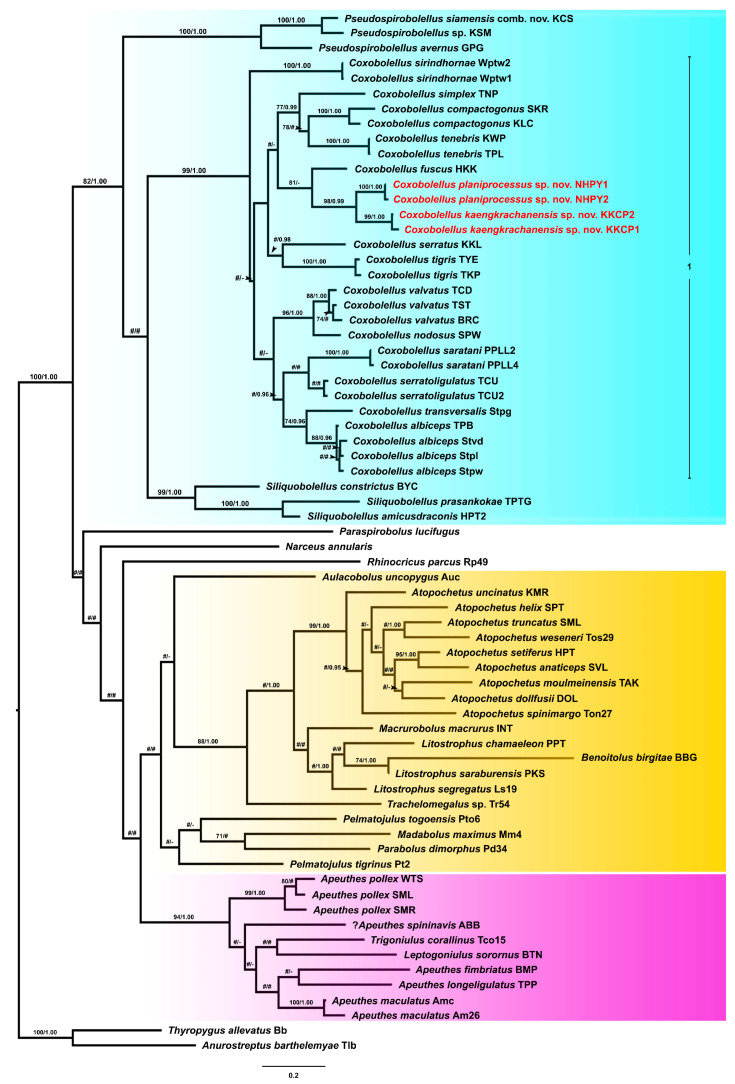
Phylogenetic relationships of *Coxobolellus* and several other spirobolidan taxa based on maximum likelihood analysis (ML) and Bayesian inference (BI) of a 660 bp of the cytochrome c oxidase subunit I gene fragment. Numbers at nodes indicate branch support based on bootstrapping (ML)/posterior probabilities (BI). Scale bar = 0.2 substitutions/site. # indicates branches with <70% ML bootstrap support or <0.95 posterior probability, - indicates non-supported branches. The coloured areas mark the Trigoniulinae (purple), Pseudospirobolellidae (blue), and non-trigoniuline Pachybolidae (yellow). The *Coxobolellus* clade (1) is marked by a vertical line.

The ML and BI trees constructed from the COI, 16S rRNA, and combined datasets showed highly congruent topologies with respect to the well-supported branches. The phylogenetic trees based on the single 16S rRNA dataset and the combined dataset (COI+16S rRNA) are presented in the Supplementary Material (Figures S1 and S2, respectively), whereas the COI tree is used for further discussion (Figure 1).

The trees consistently showed that (Figure 1): (1) the genera *Coxobolellus*, *Pseudospirobolellus*, and *Siliquobolellus* form a well-supported Pseudospirobolellidae clade (BV = 82; PP = 1.00) that includes *P. siamensis* comb. nov. as sister group of *Pseudospirobolellus* sp. KSM from Koh Samed (BV = 100; PP = 1.00), (2) the genus *Coxobolellus* forms a well-supported clade (blue clade 1 in Figure 1) (BV = 99; PP = 1.00) that includes *C. kaengkrachanensis* sp. nov. and *C. planiprocessus* sp. nov. as well-supported sister taxa (BV = 98; PP = 0.99) together with *C. fuscus* Pimvichai, Enghoff, Panha & Backeljau, 2020 (BV = 81; no PP support), and (3) the COI sequence of *Benoitolus birgitae* appears as a long branch that does not cluster with *P. siamensis* comb. nov., but that is positioned with high support in the pachybolid genus *Litostrophus*.

### 3.2. Species Delimitation Based on COI Sequences

#### 3.2.1. ASAP

Fifteen molecular operational taxonomic units (MOTUs) were found with a probability of 1.00 and with a recursive split *p*-value of 0.01 (Table 1). 

#### 3.2.2. GMYC

With both the single and multiple thresholds, the ML values of the null model (=all sequences belong to a single species) were lower than the ML value of the GMYC model (Table 2). The single threshold option yielded nine clusters and 16 MOTUs with confidence intervals (CI) ranging from 3 to 20 (Table 1). The multiple thresholds option yielded nine clusters and 18 MOTUs with CI ranging from 13 to 18 (Table 1).

#### 3.2.3. PTP

Species delimitation results from PTP suggest 16 MOTUs (Table 1), as for GMYC single threshold (estimated number of MOTUs is between 13 and 22, mean: 17.43; Table 1).

### 3.3. Taxonomy

Class DIPLOPODA de Blainville in Gervais, 1844.

Order SPIROBOLIDA Bollman, 1893.

Suborder SPIROBOLIDEA Bollman, 1893.

Family PSEUDOSPIROBOLELLIDAE Brölemann, 1913.

Genus *Pseudospirobolellus* Carl, 1912.

The genus *Pseudospirobolellus* currently comprises the following three species:

*P. avernus* (Butler, 1876).

*P. siamensis* (Attems, 1936) comb. nov.

*P. sigmoides* Attems, 1953.

### 3.4. Redescription of Pseudospirobolellus siamensis (Attems, 1936) comb. nov.

**Figure 2:**
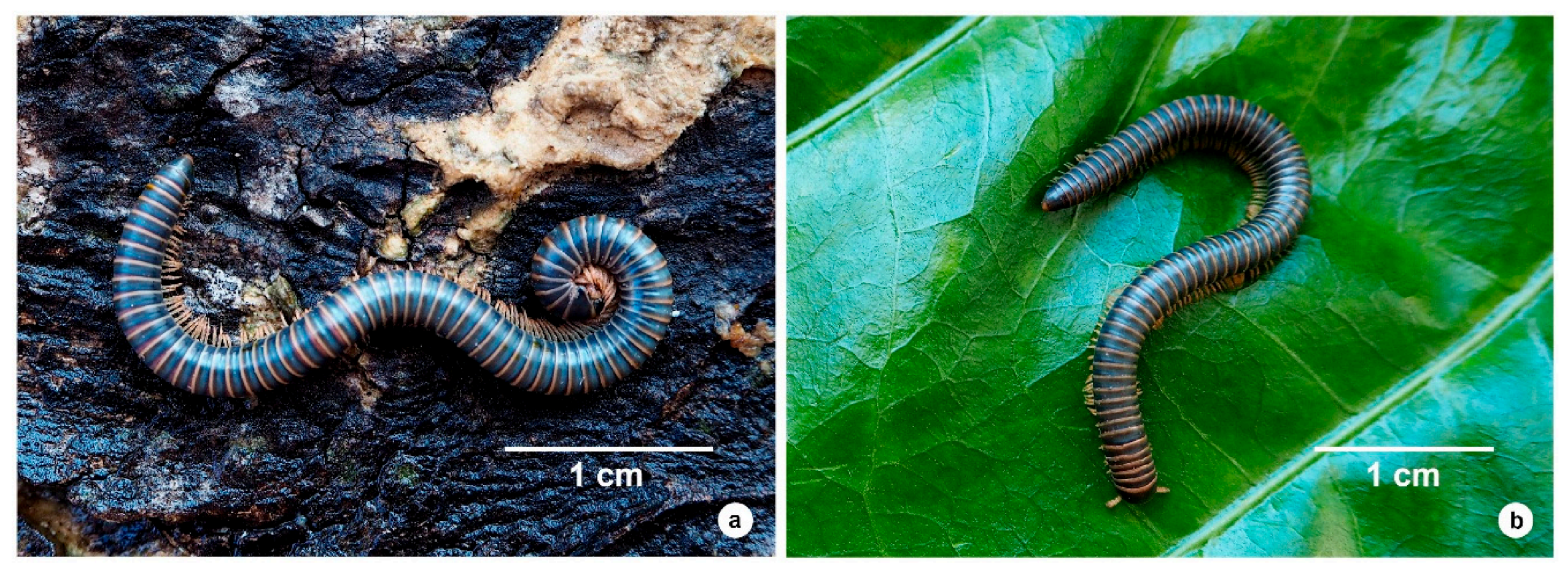
Live *Pseudospirobolellus siamensis* comb. nov. from Koh Samae San, Sattahip District, Chonburi Province, Thailand (CUMZ-D00160). (**a**): living *P. siamensis* comb. nov. with the body partially curled; (**b**): *P. siamensis* comb. nov. while walking.

**Figure 3:**
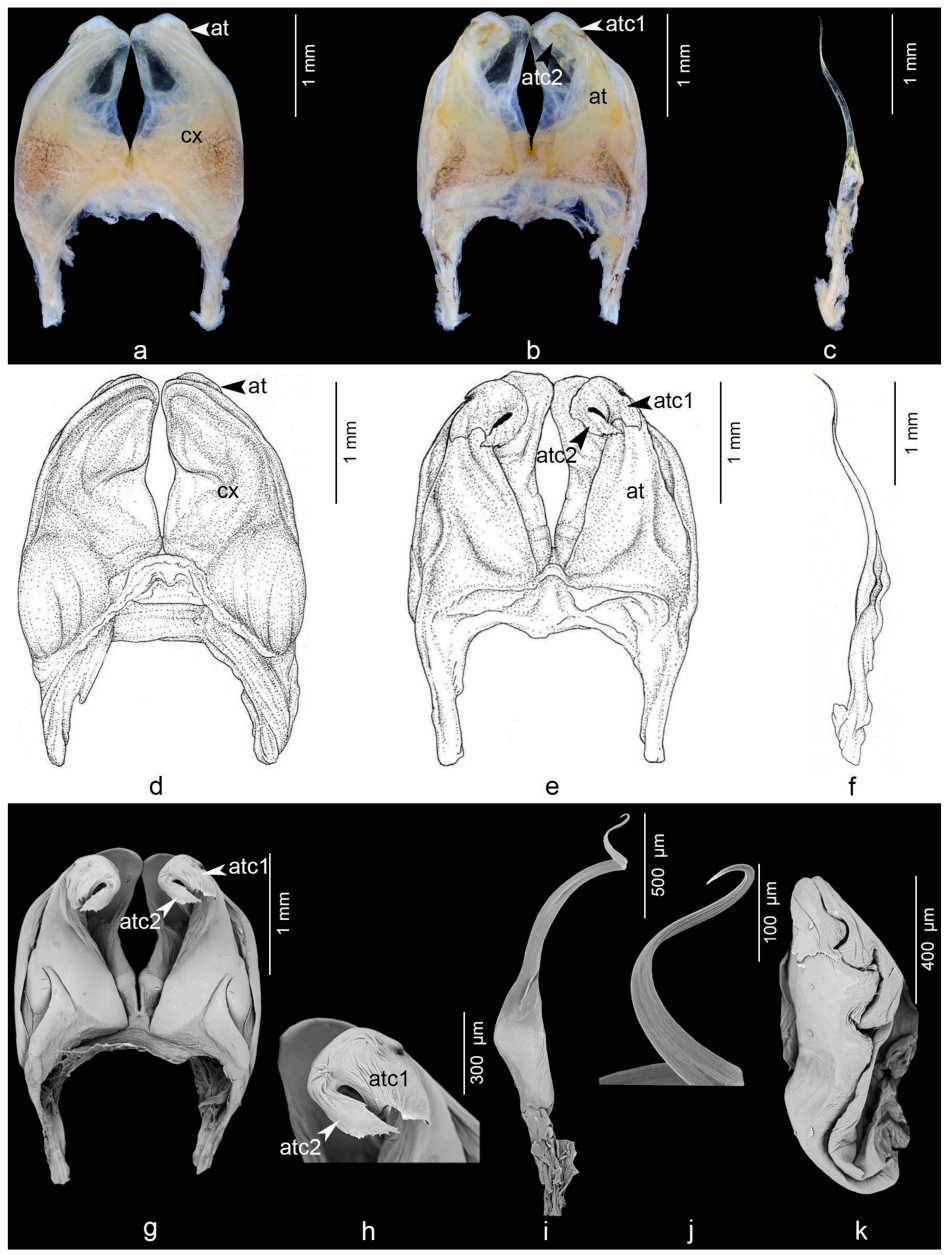
*Pseudospirobolellus siamensis* comb. nov., gonopods. (**a**–**f**,**i**,**j**): specimen from Koh Samae San, CUMZ-D00160; (**g**,**h**,**k**): specimen from Koh Chuang, CUMZ-D00118 and CUMZ-D00118-2. (**a**) Anterior gonopod, anterior view. (**b**) Anterior gonopod, posterior view. (**c**) Right posterior gonopod, lateral view. (**d**) Anterior gonopod, anterior view. (**e**) Anterior gonopod, posterior view. (**f**) Right posterior gonopod, lateral view. (**g**) SEM, anterior gonopod, postero-mesal view. (**h**) SEM, tip of left anterior gonopod. (**i**) SEM, left posterior gonopod, latero-mesal view. (**j**) SEM, tip of left posterior gonopod, postero-mesal view. (**k**) left vulva, postero-lateral view. For abbreviations see Section 2.2.

**Figure 4:**
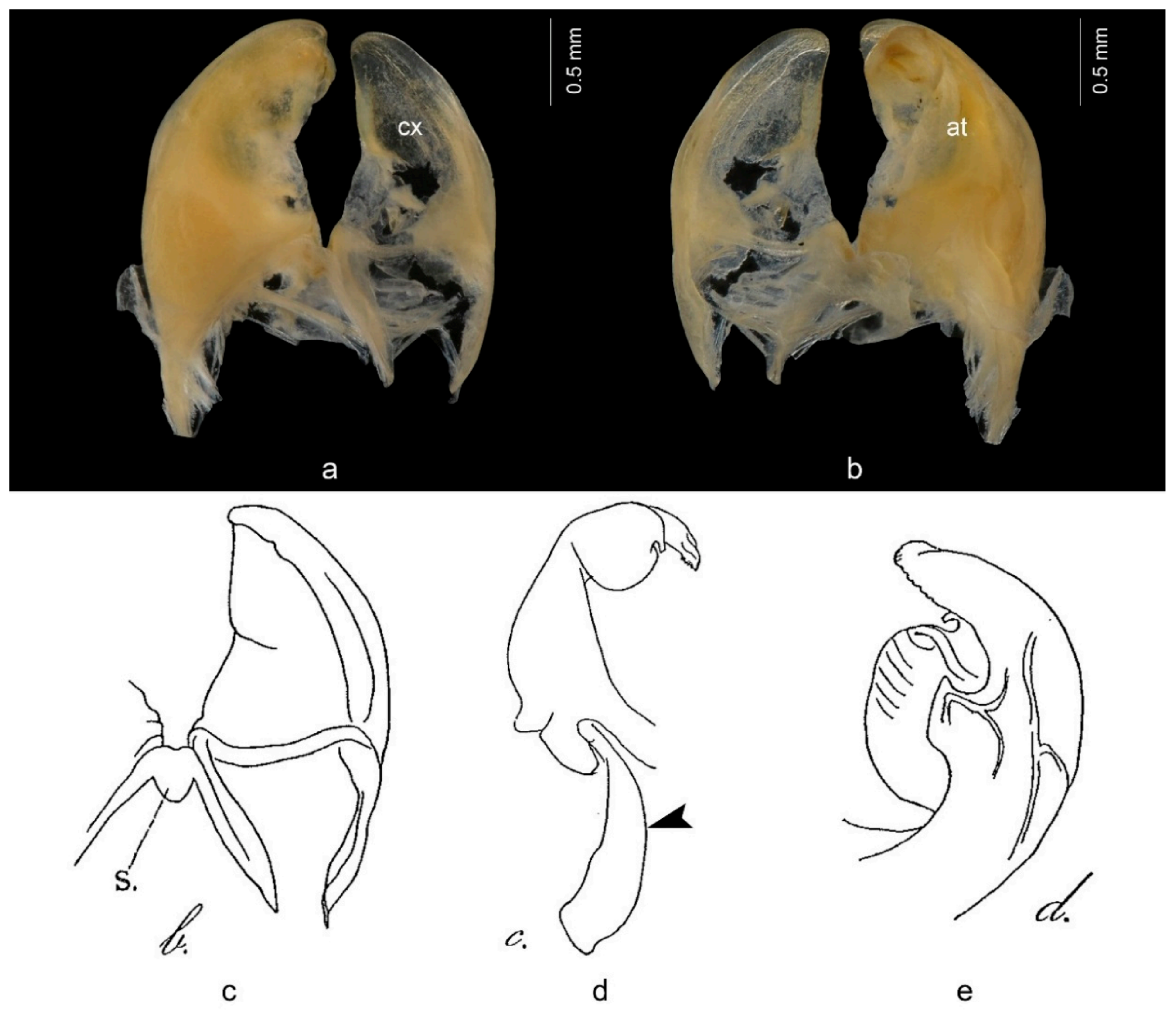
*Pseudospirobolellus siamensis* comb. nov., holotype gonopods (from Koh Samae San, NHMW-Inv. No. 2345). (**a**) Anterior gonopod, anterior view. (**b**) Anterior gonopod, posterior view. (**c**–**e**) Original figures 85 b–d of Attems (1936) [30], with the original captions in parentheses. (**c**) Anterior gonopod (“b. anterior gonopod, S. sternite”). (**d**) Right anterior gonopod telopodite (“c. d. posterior gonopod”). (**e**) Left anterior gonopod telopodite (“c. d. posterior gonopod”). For abbreviations see Section 2.2.

(Figure 2, Figure 3 and Figure 4)

*Cyclothyrophorus siamensis* (Attems, 1936: 300).

*Benoitolus siamensis* (Attems, 1936).

**Material Studied**

**Holotype.** 1 male (NHMW-Inv. No. 2345, Table S1)), Thailand, Chonburi Province, Sattahip District, Koh Samae San; October 1916; C. Boden Kloss leg. 

**Other Material Studied.** 1 male (CUMZ-D00160), 3 females (CUMZ-D00160-1), Thailand, Chonburi Province, Sattahip District, Koh Samae San; 12°33′58″ N, 100°57′02″ E; 7 m a.s.l.; 3 October 2019; P. Pimvichai leg. 1 male (CUMZ-D00118), 3 females (CUMZ-D001118-2), Thailand, Chonburi Province, Sattahip District, Koh Chuang; 12°31′45″ N, 100°57′17″ E; 9 m a.s.l.; 21 July 2014; P. Pimvichai leg.

**Species description.** Adult males with 64–66 podous rings, length ca. 5–6 cm, diameter ca. 3.5–4.1 mm. Adult females with 61–63 podous rings, length ca. 5–6 cm, diameter ca. 4.1–4.3 mm. No apodous rings in front of telson.

**Colour.** Living animal dark gray. Antennae, legs, edge of collum, metazona, and preanal process light orange (Figure 2a,b).

**Head capsule.** Smooth. Occipital furrow extending down between, but not beyond eyes; clypeal furrow reaching level of antennal sockets. Area below antennal sockets and eyes impressed, forming part of antennal furrow. Incisura lateralis open. 2 + 2 labral teeth, a row of 5 + 5 supralabral setae. Diameter of eyes ca. half of interocular space; 9–10 vertical rows of ommatidia, 5 horizontal rows, 41–43 ommatidia per eye. Antennae short, not reaching beyond collum when stretched back, accommodated in a shallow furrow composed of a horizontal segment in the head capsule and a vertical segment in the mandibular cardo and stipes. Antennomere lengths 2 > 6 > 3 = 5 > 4 > 1 > 7; antennomere 1 glabrous, 2, 3 and 4 with some ventral setae, 5, 6 and 7 densely setose; 4 apical sensilla. Mandibles: Stipes broad at the base and gradually narrowing towards the apex, forming a triangular shape. Gnathochilarium: each stipes with 2 or 3 apical setae; each lamella lingualis with 2 setae, one behind the other. Basal part of mentum transversely wrinkled; basal part of stipes longitudinally wrinkled.

**Collum.** Smooth, with a marginal furrow along lateral part of anterior margin; lateral lobes narrowly rounded, extending as far ventrad as the ventral margin of body ring 2.

**Body rings.** Rings 2–5, ventrally concave, hence with distinct ventrolateral corners. Body rings very smooth, sides parallel in dorsal view. Tergo-pleural suture visible on pro- and mesozona. Prozona smooth; mesozona ventrally with fine oblique striae, dorsally punctate; metazona ventrally with fine longitudinal striae, otherwise smooth. Pleural parts of rings with fine oblique striae. Sterna transversely striate. Ozopores from ring 6, situated in mesozona, ca. 1 pore diameter in front of metazona. Sutures between pro-, meso- and metazona distinct; horizontal suture distinct on pro-, meso- and metazona.

**Telson.** Smooth, preanal ring with slightly concave dorsal profile, with a short process protruding as far as, or slightly beyond, anal valves. Anal valves smooth, rounded. Subanal scale broadly triangular.

**Legs.** Length of midbody legs 71% of body width in males, 47–49% of body width in females. Prefemur basally constricted and longer than other podomeres. First and second legs with 2 prefemoral, 2 femoral, 2 postfemoral, and 2 tibial setae, and 2–3 ventral apical setae on tarsi, numbers of setae reaching constancy from pair 3: each leg with 1 coxal, 1 femoral, 1 postfemoral, 1 tibial seta, tarsi with 2–3 ventral and one dorsal apical seta; females with 1 coxal, 1 prefemoral, 1 femoral, 1 postfemoral, 1 tibial seta, tarsi with 1–3 ventral and one dorsal apical seta, the apical ventral seta larger than the more basal one. In males, coxa of 5th legs with a large thick rounded process, large ventral pads on prefemur.

**Anterior gonopods** (Figure 3a,b,d,e,g,h)**.** Coxae (cx) very high, approximately equal in height to telopodites (at); broad at base, slightly narrowing distally, apically rounded and gently curved mesad. Posterior surface with a distinct, relatively high lateral ridge accommodating the telopodite. Telopodites (at) subequal in height to coxae, broad basally, gradually narrowing distally, apically rounded, curved laterad, forming a canopy-like structure (atc); distally divided into 2 processes; the outer one broad (atc1); the inner one flattened with serrate margin (atc2).

**Posterior gonopods** (Figure 3c,f,i,j)**.** Simple, very slender and elongate; apically slightly sigmoid, pointed, and directed distad.

**Vulvae** (Figure 3k)**.** Valves prominent, of equal size; with two fairly large teeth, valves fitting tightly together, no trace of operculum.

**Remark.** Attems (1936: 300-301, figure 85a–d) [30] described the gonopods of this species as follows: “Sternite (S) of anterior gonopods small; anterior gonopods consisting of a hollowed-out, distally narrowed and rounded lamella; posterior gonopod not distinctly segmented, of a broad sickle-like shape, at the tip a broad rounded lamella and a triangular tooth”.

However, Attems’s (1936: figure 85c,d) [30] drawings of the posterior gonopod telopodite actually show the anterior gonopod telopodite (Figure 4d,e), while the posterior gonopod telopodite appears to be faintly indicated as a small, elongate basal element hidden inside the anterior gonopod telopodite, as shown at the basal part in Figure 4d, arrow (=figure 85c of Attems, 1936) [30].

### 3.5. Genus Coxobolellus Pimvichai, Enghoff, Panha & Backeljau, 2020

The genus *Coxobolellus* currently comprises the following 15 species:

*C. albiceps* Pimvichai, Enghoff, Panha & Backeljau, 2020.

*C. compactogonus* Pimvichai, Enghoff, Panha & Backeljau, 2020.

*C. fuscus* Pimvichai, Enghoff, Panha & Backeljau, 2020.

*C. nodosus* Pimvichai, Enghoff, Panha & Backeljau, 2020.

*C. serratus* Pimvichai, Enghoff, Panha & Backeljau, 2020.

*C. simplex* Pimvichai, Enghoff, Panha & Backeljau, 2020.

*C. tenebris* Pimvichai, Enghoff, Panha & Backeljau, 2020.

*C. tigris* Pimvichai, Enghoff, Panha & Backeljau, 2020.

*C. transversalis* Pimvichai, Enghoff, Panha & Backeljau, 2020.

*C. valvatus* Pimvichai, Enghoff, Panha & Backeljau, 2020.

*C. saratani* Pimvichai, Enghoff, Panha & Backeljau, 2022.

*C. serratoligulatus* Pimvichai, Enghoff, Panha & Backeljau, 2022.

*C. sirindhornae* Pimvichai, Prasankok & Backeljau, 2025.

*C. kaengkrachanensis* sp. nov.

*C. planiprocessus* sp. nov.

### 3.6. Descriptions of Two New Coxobolellus Species

The two new species conform to the general external morphology of the genus as outlined in the generic description and exhibit the four diagnostic synapomorphies that define the genus *Coxobolellus* [6,9]:

(1) A protruding process on the coxae of the 3rd (and sometimes 4th) pair of male legs;
(2) Large, triangular coxae on the 4th and 5th pairs of legs;
(3) A short process on the preanal ring extending to or slightly beyond the anal valves;
(4) The posterior gonopod telopodite divided into two parts, with a conspicuous pore opening (oeg) at the mesal margin at the end of the coxal part.

Only species-specific diagnostic characters are further described in detail.

### 3.7. Coxobolellus kaengkrachanensis sp. nov.

**Figure 5:**
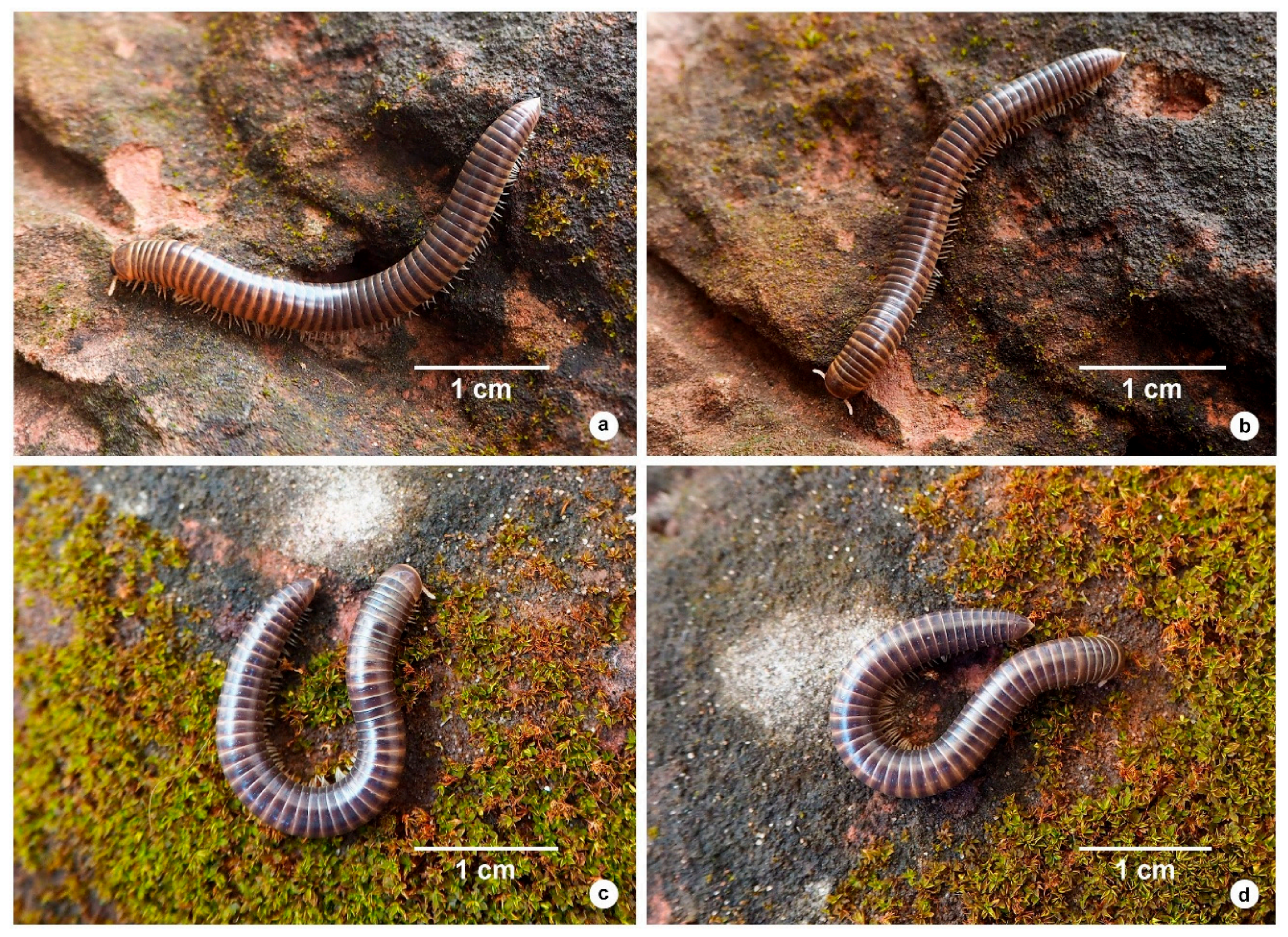
Live *Coxobolellus* species from Thailand. (**a**,**b**) *Coxobolellus kaengkrachanensis* sp. nov. (Kaeng Krachan), male (holotype, CUMZ-D00158). (**c**,**d**) *Coxobolellus planiprocessus* sp. nov. (Huai Yang waterfall), male (holotype, CUMZ-D00159).

**Figure 6:**
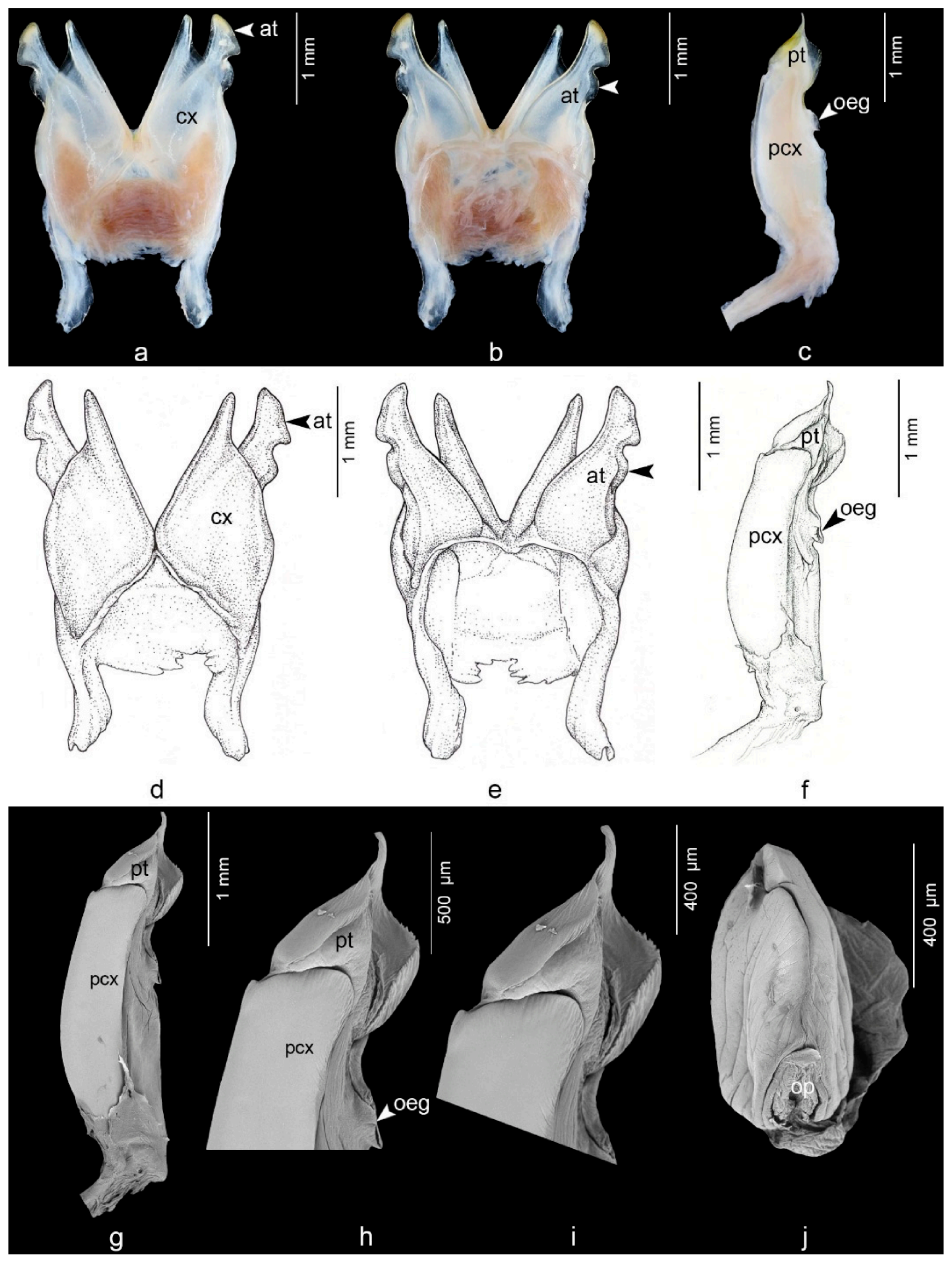
*Coxobolellus kaengkrachanensis* sp. nov., holotype, gonopods (specimen from Kaeng Krachan, CUMZ-D00158). (**a**) Anterior gonopod, anterior view. (**b**) Anterior gonopod, posterior view, the unlabelled arrow indicates the distinct lobe. (**c**) Right posterior gonopod, lateral view. (**d**) Anterior gonopod, anterior view. (**e**) Anterior gonopod, posterior view, the unlabelled arrow indicates the distinct lobe. (**f**) Right posterior gonopod, lateral view. (**g**) SEM, right posterior gonopod, postero-mesal view. (**h**) SEM, right posterior gonopod postero-mesal view. (**i**) SEM, apical part of right posterior gonopod, postero-mesal view. (**j**) SEM, left vulva, postero-mesal view. For abbreviations see Section 2.2.

(Figure 5, Figure 6 and Figure 8 (droplet no. 14))

**Material Studied**

**Holotype.** 1 male (CUMZ-D00158), Thailand, Phetchaburi Province, Kaeng Krachan District, Kaeng Krachan; 12°47′54″ N, 99°27′37″ E; 310 m a.s.l.; 11 November 2022; P. Pimvichai, B. Segers, K. Breugelmans and T. Backeljau leg.

**Paratypes.** 1 male (CUMZ-D00158-2), 3 females (CUMZ-D00158-3); same data as for holotype.

**Etymology.** The specific epithet refers to Kaeng Krachan, the type locality of this species.

**Diagnosis.** As for the genus but differing in the following details. Mesal margins of anterior gonopod straight, diverging, delimiting a V-shaped space between both coxae. Similar in this respect to *C. nodosus*, *C. saratani*, and *C. tranversalis*. Differing from all other *Coxobolellus* species mentioned by this paper by having a posterior gonopod telopodite apically ending in a coarsely serrate lamella with a sharply pointed spine.

**Description.** Adult males with 52 or 53 rings, with 1–2 apodous rings. Length ca. 5–6 cm, diameter ca. 5.0–5.2 mm. Adult females with 51–53 rings, with 2–3 apodous rings. Length ca. 5 cm, diameter ca. 5.5–5.6 mm.

**Colour.** Living animal dark brown. Antennae, legs, edge of collum, metazona, and preanal process whitish brown (Figure 5a,b).

**Anterior gonopods** (Figure 6a,b,d,e)**.** With very high coxae (cx), as high as anterior gonopod telopodite, gradually narrowing towards tip, apically slender, narrow, mesal margins straight, diverging, delimiting a V-shaped space between both coxae; at base of posterior surface with fairly high ridge laterally for accommodation of telopodite. Telopodite far overreaching coxa, lateral margins of anterior gonopod telopodite (at) with two distinct constrict surface at 1/3 and 2/3 of its height, forming a distinct lobe (Figure 6b,e, unlabelled arrow), apically forming a triangular process.

**Posterior gonopods** (Figure 6c,f–i)**.** Simple, with very long, smooth coxal part (pcx); telopodital part (pt) short, curving mesad, apically ending in a coarsely serrate lamella with a sharply pointed spine.

**Vulvae** (Figure 6j)**.** Valves prominent, of equal size, with triangular operculum (op) at laterobasal end of vulva.

**DNA barcodes.** The GenBank accession number of the COI barcode of the holotype is PZ310494 (voucher code CUMZ-D00158; sequence isolate KKCP1) and for the paratype is PZ310495 (voucher code CUMZ-D00158-1; sequence isolate KKCP2).

**Habitat.** Found in a rotten log.

**Distribution.** Known only from the type locality, Kaeng Krachan in Phetchaburi Province, Thailand (Figure 8, droplet no. 14).

### 3.8. Coxobolellus planiprocessus sp. nov.

**Figure 7:**
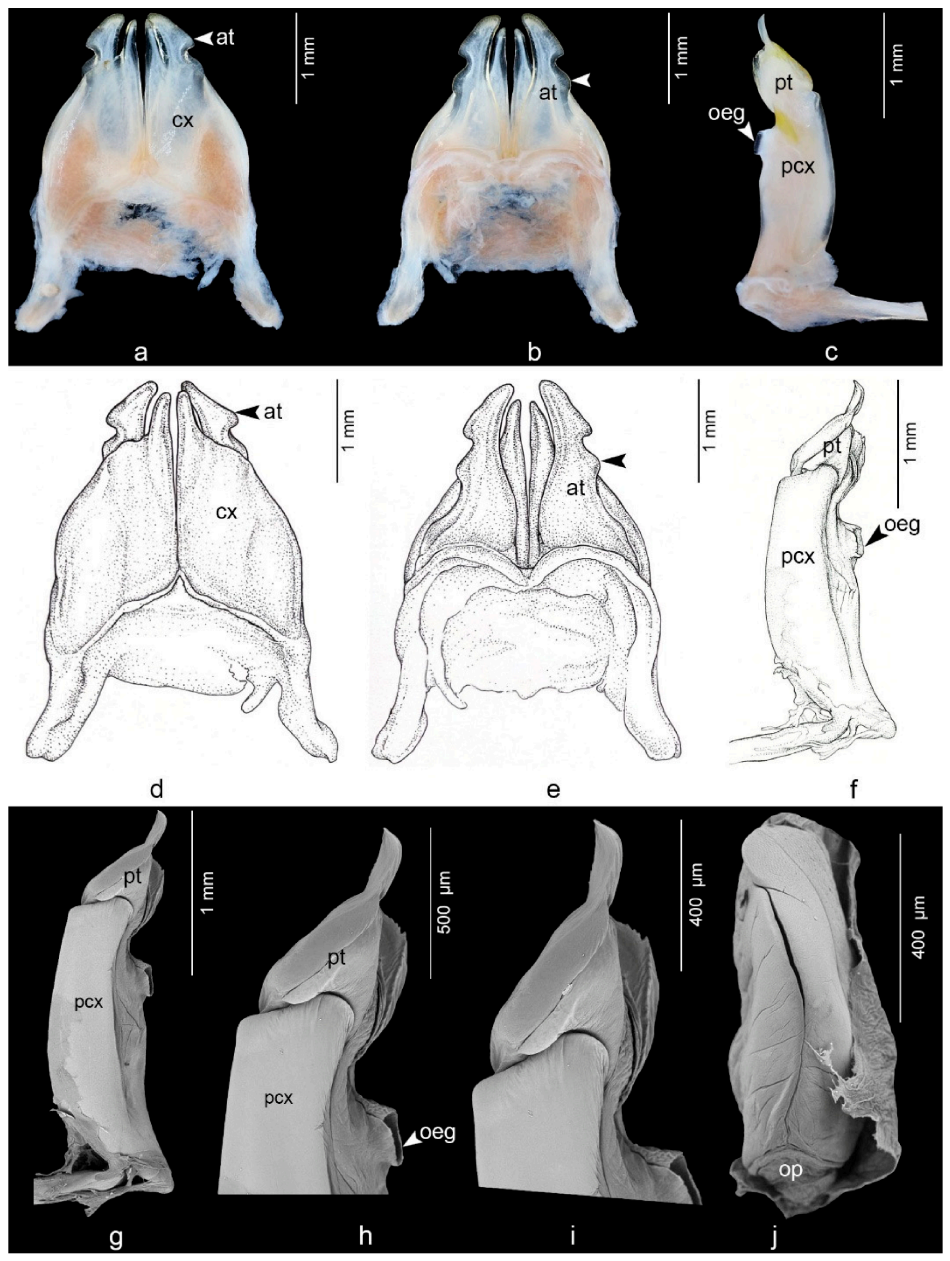
*Coxobolellus planiprocessus* sp. nov., holotype, gonopods (specimen from Huai Yang waterfall, CUMZ-D00159). (**a**) Anterior gonopod, anterior view. (**b**) Anterior gonopod, posterior view, unlabelled arrow indicates the distinct lobe. (**c**) Left posterior gonopod, lateral view. (**d**) Anterior gonopod, anterior view. (**e**) Anterior gonopod, posterior view, unlabelled arrow indicates the distinct lobe. (**f**) Right posterior gonopod, lateral view. (**g**) SEM, right posterior gonopod, postero-mesal view. (**h**) SEM, right posterior gonopod, postero-mesal view. (**i**) SEM, Apical part of right posterior gonopod, postero-mesal view. (**j**) SEM, left vulva, postero-mesal view. For abbreviations see Section 2.2.

**Figure 8:**
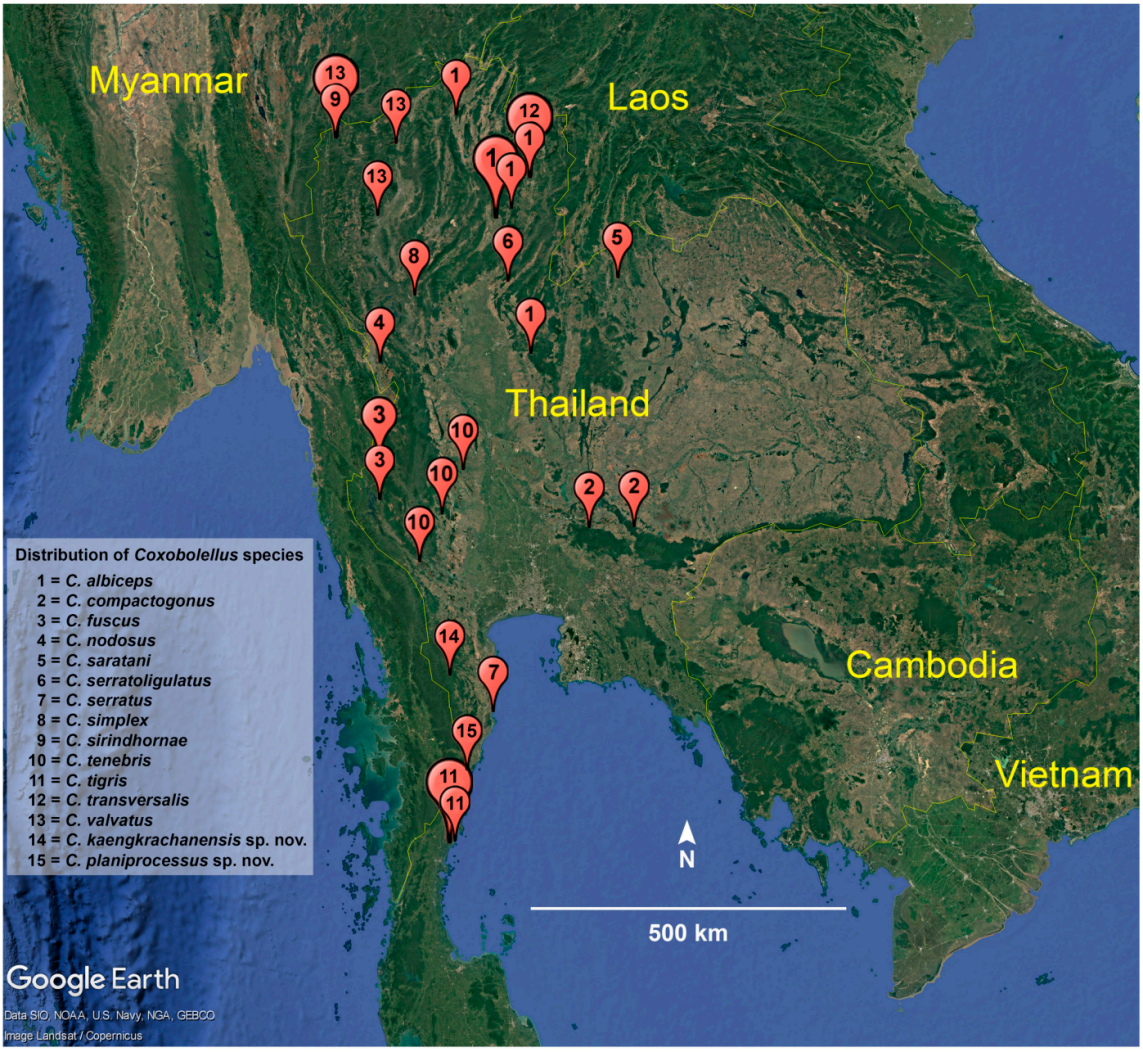
Distribution of the species of *Coxobolellus* in Thailand. Droplets vary in size only to improve readability.

(Figure 5, Figure 7 and Figure 8 (droplet no. 15))

**Material Studied**

**Holotype**. 1 male (CUMZ-D00159), Thailand, Prachuap Khiri Khan Province, Thap Sakae District, Huai Yang Waterfall; 11°37′31″ N, 99°36′54″ E; 80 m a.s.l.; 12 November 2022; P. Pimvichai, B. Segers, K. Breugelmans and T. Backeljau leg.

**Paratypes.** 8 males (CUMZ-D00159-1), 5 females (CUMZ-D00159-2); same data as for holotype.

**Etymology.** The specific epithet *planiprocessus* (Latin *planus* = flat; *processus* = projection) refers to the distinctly flattened process of the tip of the posterior gonopod telopodite, a diagnostic character of the species.

**Diagnosis.** As for the genus but differing in the following details. Anterior gonopods with very high coxae, gradually narrowing towards tip, apically slender, narrow. Similar in this respect to *C. fuscus*, *C. simplex*, *C. tenebris* and *C. kaengkrachanensis* sp. nov. Differing from all other *Coxobolellus* species mentioned in this paper by having the tip of the telopodital part (pt) of the posterior gonopod ending in coarsely serrate lamella with a long, flattened, distad directed process.

**Description.** Adult males with 50–53 rings, with 2–4 apodous rings. Length ~4–5 cm, diameter 3.9–4.0 mm. Adult females with 51–53 rings, with 2–4 apodous rings. Length ~5 cm, diameter 4.9–5.0 mm.

**Colour.** Living animal dark brown. Antennae, legs, edge of collum, metazona, and preanal process whitish brown (Figure 5c,d).

**Anterior gonopods** (Figure 7a,b,d,e)**.** With very high coxae, gradually narrowing towards tip, apically slender, narrow, mesal margin straight; at base of posterior surface with fairly high ridge laterally for accommodation of telopodite. Telopodite far overreaching coxa, apically abruptly narrow, curving mesad, lateral margins of anterior gonopod telopodite (at) distinctly constricted surfaces at 1/3 and 2/3 of its height, forming a distinct lobe (Figure 7b,e, unlabelled arrow), apically forming a narrow, triangular process.

**Posterior gonopods** (Figure 7c,f–i)**.** Simple, with very long, smooth coxal part (pcx); telopodital part (pt) short, curving mesad, apically ending in a coarsely serrate lamella with a long, flattened, distad directed process.

**Vulvae** (Figure 7j)**.** Valves prominent, of equal size, with triangular operculum (op) at laterobasal end of vulva.

**DNA barcodes.** The GenBank accession number of the COI barcode of the holotype is PZ310496 (voucher code CUMZ-D00159; sequence isolate NHYP1) and for the paratype is PZ310497 (voucher code CUMZ-D00159-1; sequence isolate NHYP2).

**Habitat.** Found under leaf litter on limestone rock formations.

**Distribution.** Known only from the type locality Huai Yang waterfall in Prachuap Khiri Khan Province, Thailand (Figure 8, droplet no. 15).

## 4. Discussion

Morphologically, the two new *Coxobolellus* species can be confidently assigned to this genus as they show its four diagnostic synapomorphic characters (see Section 3.6). At the same time, the two new species differ from all other *Coxobolellus* species mentioned in this paper by the structure of the posterior gonopod telopodite, with *C. kaengkrachanensis* sp. nov. having a telopodite that apically terminates in a coarsely serrate lamella bearing a sharply pointed spine, whereas *C. planiprocessus* sp. nov. possesses a telopodite that ends in a coarsely serrate lamella with a long, flattened, distally directed process. Furthermore, the two new species can be readily distinguished from each other by the configuration of the anterior gonopods. In *C. kaengkrachanensis* sp. nov., the mesal margins are straight and diverging, forming a distinct V-shaped space between the coxae, while in *C. planiprocessus* sp. nov. the mesal margins are also straight, but the coxae are positioned closely to each other.

The DNA sequence analyses provide additional, convincing, support for the recognition of the two new species and their assignment to the genus *Coxobolellus* since they form two well-supported, but quite deeply separated sister clades that are firmly nested within the overall *Coxobolellus* clade (Figure 1). This topological pattern is evidently also reflected in the sequence divergence values since the intraspecific sequence divergences of *C. kaengkrachanensis* sp. nov. and *C. planiprocessus* sp. nov. (varying from 0.00 to 0.02 for COI, from 0.00 to 0.01 for 16S rRNA, and 0.01 for the combined COI+16S rRNA dataset) are at least 5× smaller than the mean interspecific sequence divergence between the two new species and other *Coxobolellus* species, viz. 0.14 ± 0.01 (range: 0.08–0.16) for COI and 0.15 ± 0.02 (range: 0.09–0.19) for 16S rRNA. Moreover, these interspecific divergences are perfectly in line with those among previously recognized *Coxobolellus* species (excluding the two new taxa), i.e., 0.12 ± 0.02 (range: 0.06–0.15) for COI and 0.11 ± 0.02 (range: 0.05–0.15) for 16S rRNA. Conversely, the pairwise sequence divergence between the two new species is somewhat lower, with 0.08 ± 0.00 for COI and 0.09 ± 0.00 for 16S rRNA, but still within the range of sequence divergences observed between closely related, yet morphologically well-defined, distinct millipede species, such as, for example, *C. transversalis* and *C. albiceps* (0.08 for COI and 0.07-0.10 for 16S rRNA; Tables S2 and S3) or *Thyropygus opinatus* and *T. navychula* (0.08 for COI and 0.04 for 16S rRNA [9]).

Finally, the two new *Coxobolellus* species are consistently recovered as distinct MOTUs by the three species delimitation methods, even if these methods yielded different results for some other MOTUs. The GMYC model recovered 16 putative species under a single-threshold model and 18 under a multiple-threshold model, whereas ASAP suggested 15 species and the PTP model recovered 16 species. These discrepancies are probably due to the varying levels of intraspecific divergence within, for example, *C. compactogonus*, *C. valvatus* and *C. albiceps*, where conspecific sequence divergences may reach 0.05, i.e., a value close to the interspecific threshold of 0.06–0.07 [7,31]. Anyway, taken together, the consistent morphological differences in the gonopods, the phylogenetic positioning as two well-supported clades within *Coxobolellus*, the high interspecific DNA sequence divergences, and the consistent separation as MOTUs by ASAP, GMYC and PTP, strongly support the recognition of *C. kaengkrachanensis* sp. nov. and *C. planiprocessus* sp. nov. as two well-defined, separate species under at least the morphological, biological, phylogenetic and lineage species concepts. As such they increase the actual number of *Coxobolellus* species to 15, for which an updated morphological species identification key is presented in Appendix A.

The reassignment of *Benoitolus siamensis* to the genus *Pseudospirobolellus* is supported by both morphological and DNA sequence evidence. Firstly, it is of course necessary to confirm that the structure of the anterior gonopod and the anterior gonopod telopodite of the holotype (NHMW-Inv. No. 2345) of *B. siamensis* corresponds to that of the new specimens collected from the type locality, Koh Samae San, Sattahip, Chonburi province, Thailand. Hence, there is no doubt that these specimens effectively represent *B. siamensis*.

The phylogenetic analyses of the COI and 16S rRNA sequences strongly support the transfer of *Benoitolus siamensis* to *Pseudospirobolellus*. In the phylogenetic tree (Figure 1), the specimen from Koh Chuang (one of the nine islands of the Samae San islands group), Sattahip, Chonburi, Province, forms a maximally supported clade with *P. avernus*, with aninterspecific sequence divergences of 0.14 for COI and 0.09 for 16S rRNA (Tables S2 and S3), suggesting that the two taxa are well-separated, but still closely related, species. This is confirmed by their gonopod morphology viz., the posterior gonopod which is extremely slender, sickle-shaped, and without a process.

Conversely, the interspecific sequence divergence between *P. siamensis* comb. nov. and *Pseudospirobolellus* sp. KSM is relatively low (0.06 for COI and 0.09 for 16S rRNA; Tables S2 and S3) and somewhat undecisive with respect to its interpretation as an inter- vs. intraspecific value. By way of comparison, the intraspecific sequence divergence within, for example, *Coxobolellus compactogonus* is 0.05 for COI (Table S2) and 0.04 for 16S rRNA (Table S3). Hence, an examination of the gonopod morphology will be necessary to decide whether or not *P. siamensis* comb. nov. and *Pseudospirobolellus* sp. KSM are conspecific.

The DNA sequence results are fully congruent with the revised interpretation of the gonopod morphology of *Benoitolus siamensis*. Attems (1936: 300-301, figure 85c,d) [30] believed that he illustrated the posterior gonopod telopodite. Yet, it is argued here that he in fact illustrated the anterior gonopod telopodite, whereas the true posterior gonopod telopodite remained concealed within the anterior gonopod telopodite, so that it was overlooked in the original interpretation. Hence, the posterior gonopod telopodite of *B. siamensis* is actually independent, simple, extremely slender, elongate, and without any processes, which agrees perfectly with the defining gonopod synapomorphy of the genus *Pseudospirobolellus* (Brölemann, 1914). This revised gonopod interpretation thus demonstrates that *B. siamensis* complies with the generic diagnosis of *Pseudospirobolellus* based on gonopod morphology. Consequently, the transfer of *B. siamensis* to *Pseudospirobolellus* not only resolves the erroneous homology assumptions implied by the earlier interpretations of Attems’ (1936: figure 85) [30] illustration of the gonopod structure of *B. siamensis*, but also rectifies the ill-founded conclusion that *B. siamensis* belongs to the genus *Benoitolus* and hence would be closely related to *B. birgitae* and *B. flavicollis* Mauriès, 1980 (the type species of *Benoitolus*). Indeed, the available COI sequence of *B. birgitae* does not cluster with the familiy Pseudospirobolellidae, but is firmly nested as a long branch within the pachybolid genus *Litostrophus* (Figure 1). This raises the question as to whether the genus *Benoitolus* can be maintained within the Pseudospirobolellidae or should be reassigned to the Pachybolidae. Yet, answering this question will require additional DNA sequence data, particularly from *B. flavicollis.*

## 5. Conclusions

This study highlights the value of integrating detailed morphology with phylogeny to resolve species boundaries and generic assignments in *Coxobolellus* and related genera. The two new species, *C. kaengkrachanensis* sp. nov. and *C. planiprocessus* sp. nov., are strongly supported as distinct by gonopodal morphology, phylogenetic analyses, DNA divergences, and species delimitation results. Their recognition increases the number of known *Coxobolellus* species to 15 and underscores the diversity of the group in Thailand.

The study also reassigns *B. siamensis* to *Pseudospirobolellus* based on congruent morphological and DNA data evidence. Reinterpretation of gonopodal structures corrected previous misidentifications and revealed characters consistent with *Pseudospirobolellus*, while COI and 16S rRNA phylogenies independently confirmed this placement. This transfer resolves a long-standing conflict between morphology and phylogeny and substantially revises the concept of *Benoitolus*.

Patterns of COI and 16S rRNA divergence further support the utility of these markers for species delimitation and generic assessment, although some overlap between conspecific and interspecific values suggests recent diversification in certain lineages. Overall, the results reinforce the importance of gonopodal morphology in millipede taxonomy while emphasizing the need to re-evaluate historical interpretations using integrated morphological and molecular approaches.

## Figures and Tables

**Table 1:** Numbers of species delimited by the Assemble Species by Automatic Partitioning (ASAP) method, the General Mixed Yule Coalescent (GMYC) method, and the Poisson Tree Process (PTP) method applied to the cytochrome c oxidase I (COI) dataset of the genus *Coxobolellus*. A plus sign (+) indicates that a MOTU was identified by the respective method, whereas a minus sign (−) indicates that no MOTU was identified by that method.

**Group**	**ASAP**	**GMYC** **(Single)**	**GMYC** **(Multiple)**	**PTP**
1. *C. albiceps* Stpl + *C. albiceps* TPB + *C. albiceps* Stpw + *C. albiceps* Stvd	+	+	−	+
2. *C. albiceps* Stpl + *C. albiceps* TPB	−	−	+	−
3. *C. albiceps* Stpw	−	−	+	−
4. *C. albiceps* Stvd	−	−	+	−
5. *C. compactogonus* KLC + *C. compactogonus* SKR	+	−	+	−
6. *C. compactogonus* KLC	−	+	−	+
6. *C. compactogonus* SKR	−	+	−	+
7. *C. fuscus* HKK	+	+	+	+
8. *C. nodosus* SPW	+	+	+	+
9. *C. valvatus* BRC + *C. valvatus* TST + *C. valvatus* TCD	+	+	−	+
10. *C. valvatus* BRC + *C. valvatus* TST	−	−	+	−
11. *C. valvatus* TCD	−	−	+	−
12. *C. serratoligulatus* TCU + *C. serratoligulatus* TCU2	+	+	+	+
13. *C. serratus* KKL	+	+	+	+
14. *C. simplex* TNP	+	+	+	+
15. *C. sirindhornae* Wptw1 + *C. sirindhornae* Wptw2	+	+	+	+
16. *C. tenebris* KWP + *C. tenebris* TPL	+	+	+	+
17. *C. tigris* TKP + *C. tigris* TYE	+	+	+	+
18. *C. transversalis* Stpg	+	+	+	+
19. *C. saratani* PPLL2 + *C. saratani* PPLL4	+	+	+	+
20. *C. kaengkrachanensis* sp. nov. KKCP1 + *C. kaengkrachanensis* sp. nov. KKCP2	+	+	+	+
21. *C. planiprocessus* sp. nov. NHYP1 + *C. planiprocessus* sp. nov. NHYP2	+	+	+	+

**Table 2:** Numbers of clusters and MOTUs detected within the genus Coxobolellus by the General Mixed Yule Coalescent (GMYC) method applied to the cytochrome c oxidase I (COI) dataset.

Analysis	Clusters (CI)	MOTUs (CI)	Likelihood Null	Likelihood GMYC	Likelihood Ratio	Threshold Time
Single	9 (3–10)	16 (3–20)	126.289	129.019	5.45839	−0.01703696
Multiple	9 (9–10)	18 (13–18)	126.2898	130.4848	8.39002	−0.07556588 −0.01018455 −0.00645457

**Figure A1:**
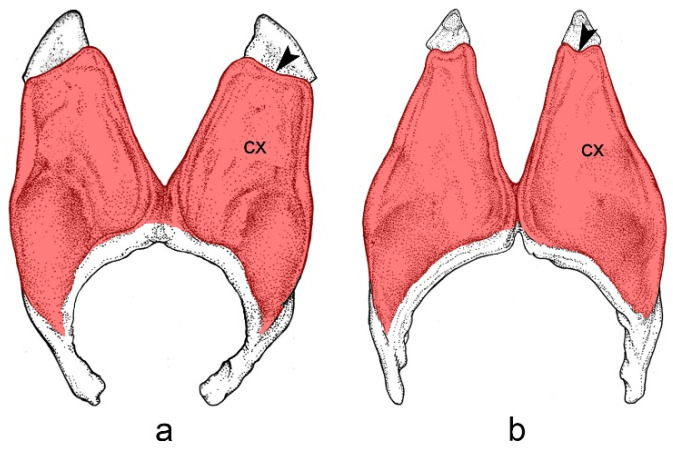
Diagnostic morphological characters used in couplet 1 of the identification key. (**a**) character supporting couplet 1a; (**b**) character supporting couplet 1b (modified from [6]).

**Figure A2:**
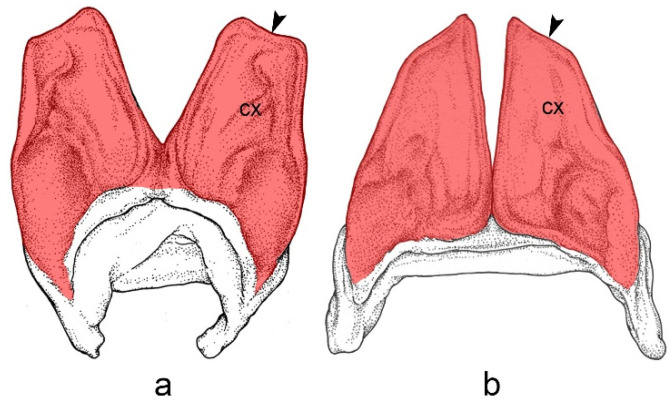
Diagnostic morphological characters used in couplet 2 of the identification key. (**a**) character supporting couplet 2a; (**b**) character supporting couplet 2b (modified from [6]).

**Figure A3:**
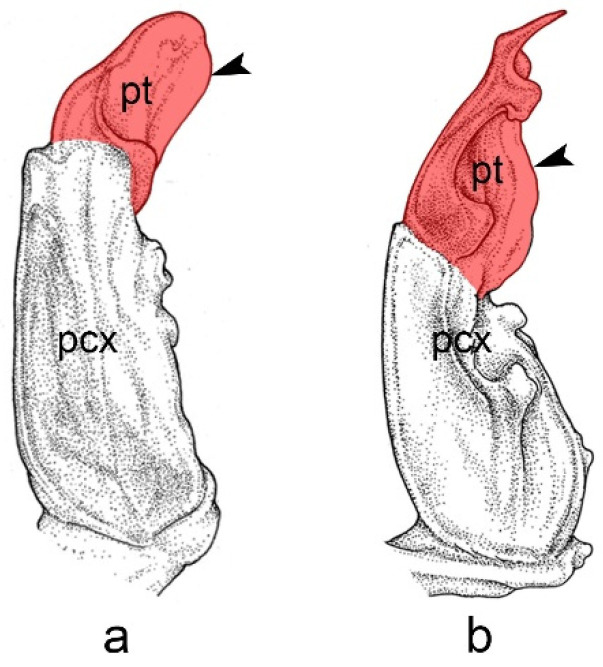
Diagnostic morphological characters used in couplet 3 of the identification key. (**a**) character supporting couplet 3a; (**b**) character supporting couplet 3b (modified from [6]).

**Figure A4:**
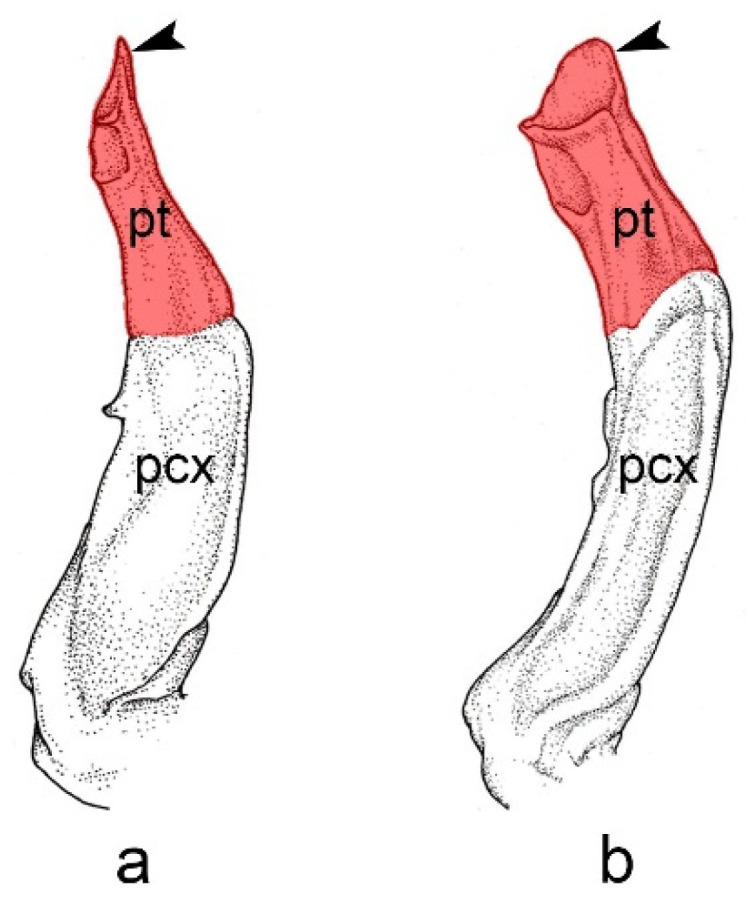
Diagnostic morphological characters used in couplet 4 of the identification key. (**a**) character supporting couplet 4a; (**b**) character supporting couplet 4b (modified from [7]).

**Figure A5:**
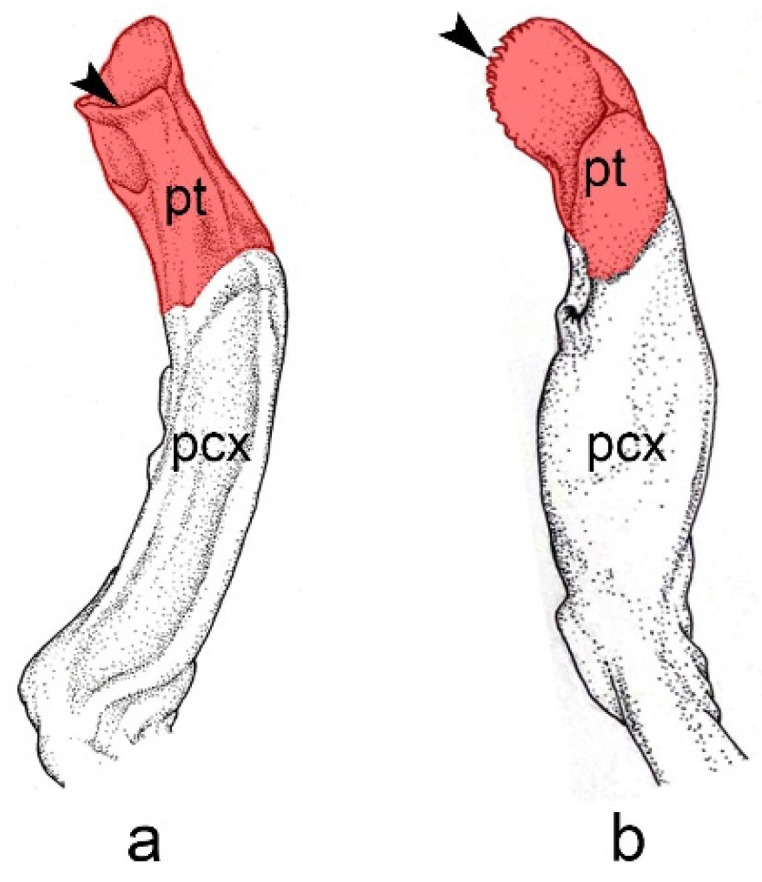
Diagnostic morphological characters used in couplet 5 of the identification key. (**a**) character supporting couplet 5a; (**b**) character supporting couplet 5b (modified from [7,9]).

**Figure A6:**
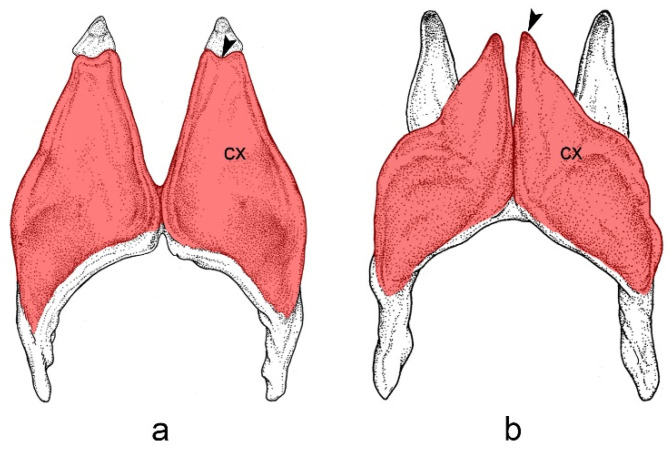
Diagnostic morphological characters used in couplet 6 of the identification key. (**a**) character supporting couplet 6a; (**b**) character supporting couplet 6b (modified from [6]).

**Figure A7:**
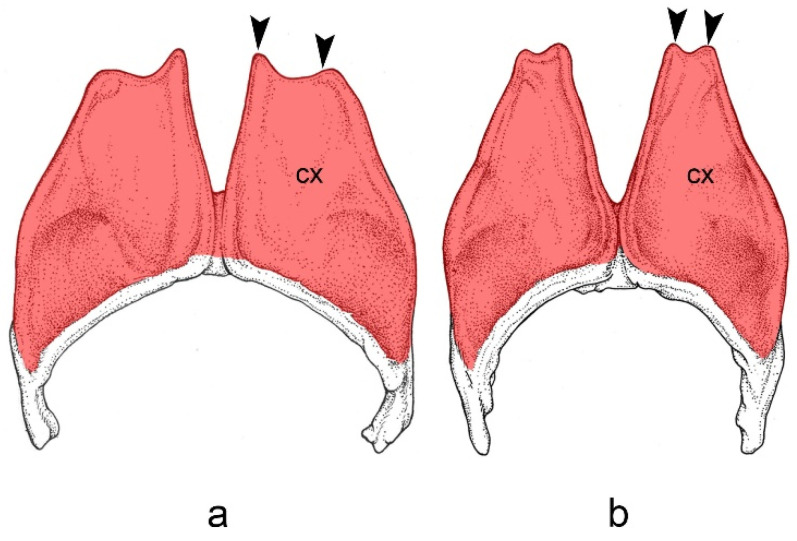
Diagnostic morphological characters used in couplet 7 of the identification key. (**a**) character supporting couplet 7a; (**b**) character supporting couplet 7b (modified from [6]).

**Figure A8:**
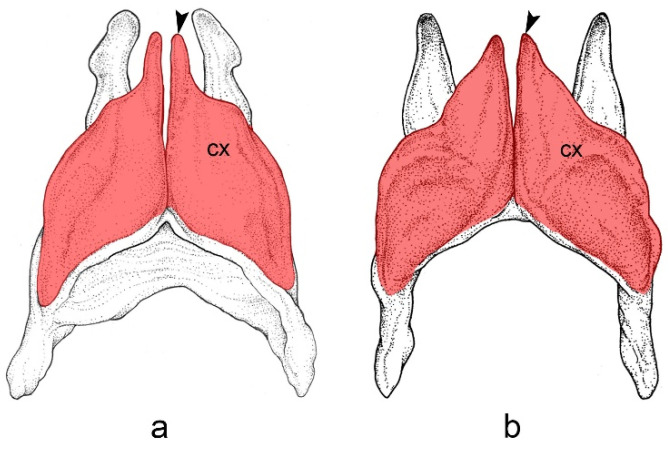
Diagnostic morphological characters used in couplet 8 of the identification key. (**a**) character supporting couplet 8a; (**b**) character supporting couplet 8b (modified from [6]).

**Figure A9:**
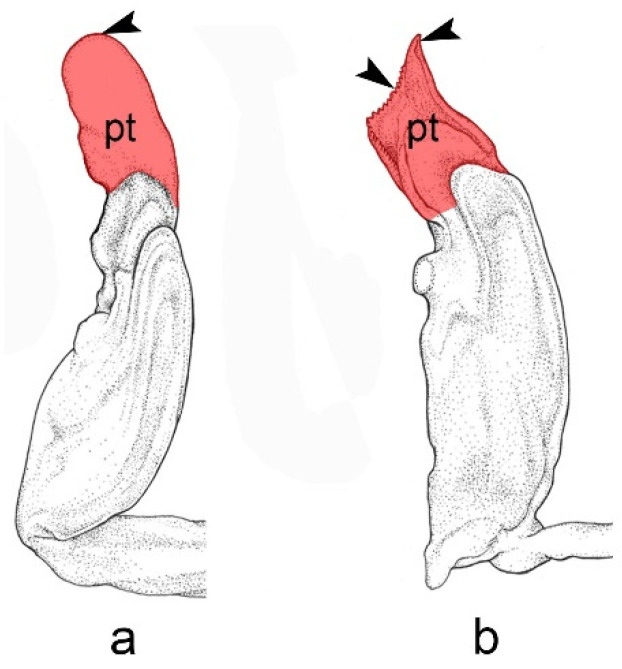
Diagnostic morphological characters used in couplet 9 of the identification key. (**a**) character supporting couplet 9a; (**b**) character supporting couplet 9b (modified from [6]).

**Figure A10:**
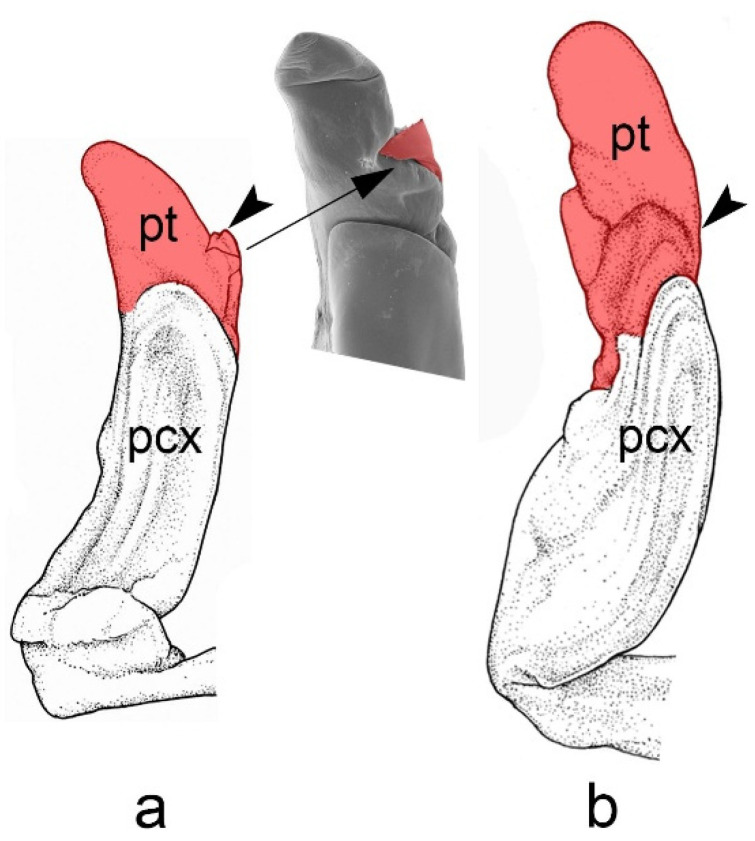
Diagnostic morphological characters used in couplet 10 of the identification key. (**a**) character supporting couplet 10a; (**b**) character supporting couplet 10b (modified from [6]).

**Figure A11:**
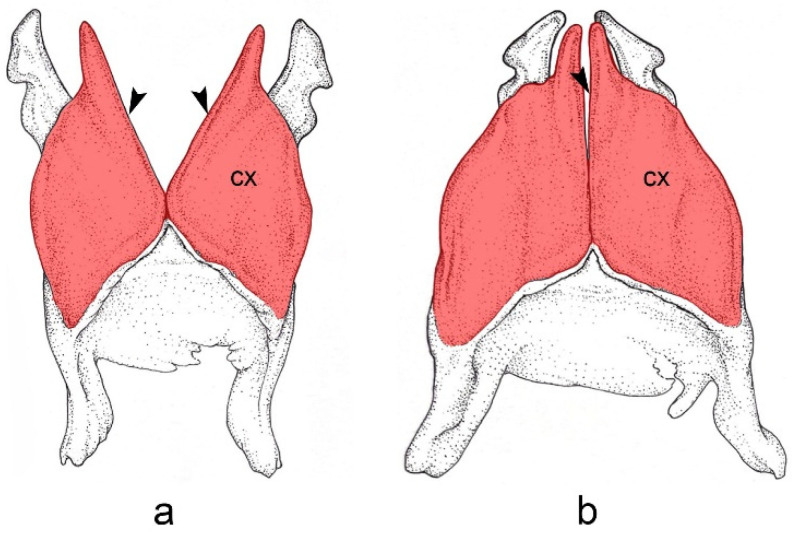
Diagnostic morphological characters used in couplet 11 of the identification key. (**a**) character supporting couplet 11a; (**b**) character supporting couplet 11b.

**Figure A12:**
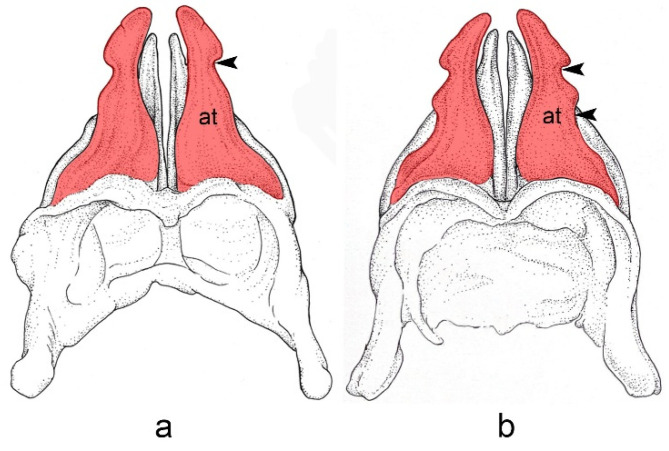
Diagnostic morphological characters used in couplet 12 of the identification key. (**a**) character supporting couplet 12a; (**b**) character supporting couplet 12b (modified from [6]).

**Figure A13:**
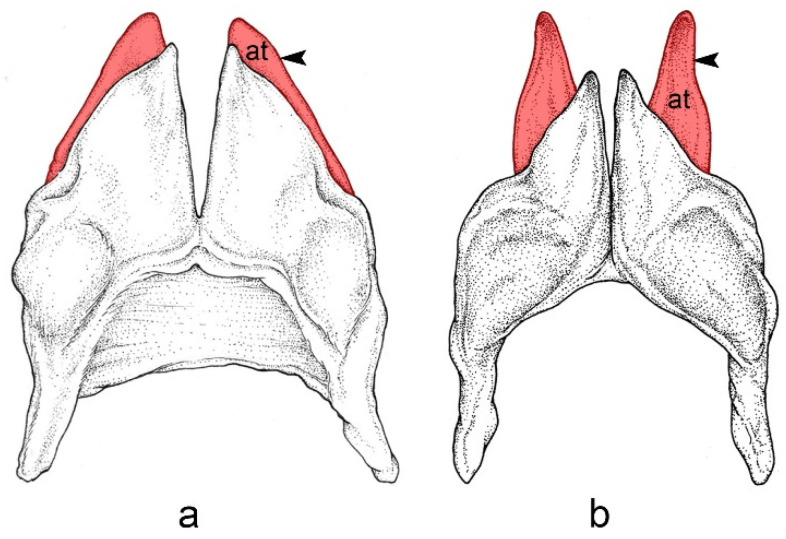
Diagnostic morphological characters used in couplet 13 of the identification key. (**a**) character supporting couplet 13a; (**b**) character supporting couplet 13b (modified from [6]).

**Figure A14:**
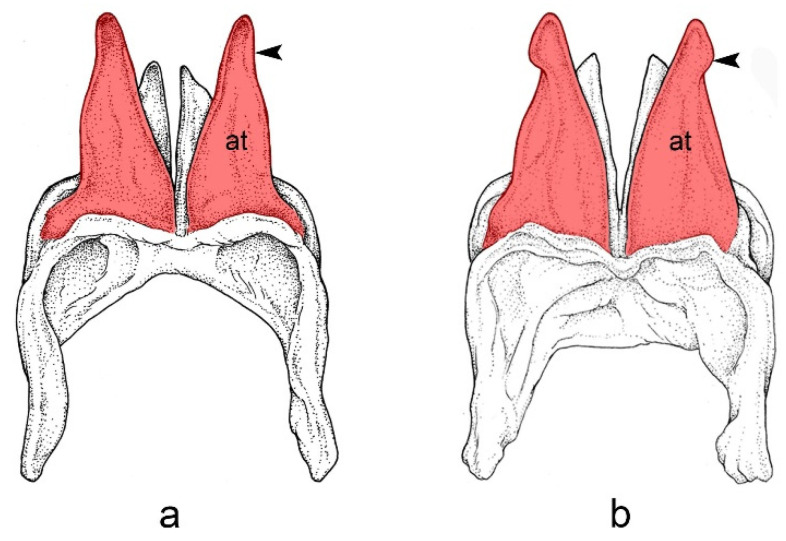
Diagnostic morphological characters used in couplet 14 of the identification key. (**a**) character supporting couplet 14a; (**b**) character supporting couplet 14b (modified from [6]).

## Data Availability

The original contributions presented in this study are included in the article/Supplementary Material. Further inquiries can be directed to the corresponding author.

## Supplementary Materials

The following supporting information can be downloaded at: https://www.mdpi.com/article/10.3390/ani16152304/s1.

## Appendix A

**Key to species of the genus *Coxobolellus* (based on adult males, update of the key by [9]** (see Section 2.2. for the abbreviations used)

1a Tip of anterior gonopod coxa truncated (Figure A1a, arrow)	2
1b Tip of anterior gonopod coxa concave/bilobed (Figure A1b, arrow) or forming a triangular process	6

2a Tip of anterior gonopod coxa transversely truncated (Figure A2a, arrow); telopodital part (pt) of posterior gonopod long compared to coxal part (pcx)	*C. transversalis*
2b Tip of anterior gonopod coxa obliquely truncated (Figure A2b, arrow); telopodital part (pt) of posterior gonopod short compared to coxal part (pcx)	3

3a Telopodital part (pt) of posterior gonopod (Figure A3a, arrow) short compared to coxal part (pcx)	*C. albiceps*
3b Telopodital part (pt) of posterior gonopod (Figure A3b, arrow) long compared to coxal part (pcx)	4

4a Telopodital part (pt) directed distad, pointed (Figure A4a, arrow), with a sharp, pointed, folded process in the middle, with a small transverse ridge near tip, with serrate mesal margin	*C*. *saratani*
4b Telopodital part (pt) curving mesad, ending in a rounded lobe (Figure A4b, arrow)	5

5a Telopodital part (pt) forming a canopy, with a broad, flattened, serrate, tongue-like process protruding from mesal surface (Figure A5a, arrow)	*C*. *serratoligulatus*
5b Telopodital part (pt) without a process protruding from mesal surface, basal part of pt with lateral margin folds mesally, apically forming a flattened, rounded lobe, with serrate mesal margin, curving mesad (Figure A5b, arrow)	*C. sirindhornae*

6a Tip of anterior gonopod coxa concave/bilobed (Figure A6a, arrow)	7
6b Tip of anterior gonopod coxa forming triangular process (Figure A6b, arrow).	8

7a Tip of anterior gonopod coxa bilobed (Figure A7a, arrows), outer process broadly rounded, inner process triangular, protruding higher than outer process; telopodital part (pt) of posterior gonopod ending in a rounded margin with a sharp spine protruding from mesal surface near tip	*C. valvatus*
7b Tip of anterior gonopod coxa concave (Figure A7b, arrows), forming equal outer and inner lobes; telopodital part of posterior gonopod (pt) ending in a long, sharp spine, with a flattened lamella protruding from mesal surface near tip	*C. nodosus*

8a Tip of anterior gonopod coxa ending in an abruptly narrowed, pointed, triangular process (Figure A8a, arrow)	9
8b Tip of anterior gonopod coxa ending in a simple triangular process (Figure A8b, arrow)	13

9a Posterior gonopods telopodite apically ending in a smooth, rounded lobe (Figure A9a, arrow)	10
9B Posterior gonopods telopodite apically ending in a serrated and/or pointed process (Figure A9b, arrows)	11

10a Telopodital part (pt) of posterior gonopod with a sharp, curling lamella at base (Figure A10a, arrow)	*C. tenebris*
10b Telopodital part (pt) of posterior gonopod without a sharp, curling lamella at base (Figure A10b, arrow)	*C. simplex*

11a Mesal margins of anterior gonopods straight, diverging, delimiting a V-shaped space between both coxae (Figure A11a, arrow); lateral margins of anterior gonopod telopodite (at) with two distinct constrict surface at 1/3 and 2/3 of its height, forming a distinct lobe (Figure 6b,e, arrow); tip of telopodital part (pt) of posterior gonopod ending in coarsely serrate lamella with a sharply pointed spine	*Coxobolellus kaengkrachanensis* sp. nov.
11b Mesal margins of anterior gonopods straight, with the coxae positioned closely together (Figure A11b, arrow)	12

12a Lateral margins of anterior gonopod telopodite (at) with slightly constrict surface at 2/3 of its height (Figure A12a, arrow); tip of telopodital part (pt) of posterior gonopod ending in coarsely serrate lamella with a sharp point spine	*C. fuscus*
12b Lateral margins of anterior gonopod telopodite (at) with two distinct constrict surface at 1/3 and 2/3 of its height (Figure A12b, arrow), forming a distinct lobe (Figure 7b,e, arrow); tip of telopodital part (pt) of posterior gonopod ending in coarsely serrate lamella with a long, flattened, distad directed process (Figure 7c,f–i)	*Coxobolellus planiprocessus* sp. nov.

13a Anterior gonopod telopodite (at) projecting slightly over anterior gonopod coxa (cx), with rounded tip (Figure A13a, arrow)	*C. compactogonus*
13b Anterior gonopod telopodite (at) far overreaching anterior gonopod coxa (cx), with narrowed tip (Figure A13b, arrow)	12

14a Anterior gonopod telopodite (at) directed distad (Figure A14a, arrow); telopodital part (pt) of posterior gonopod ending in a rounded, serrate margin	*C. tigris*
14b Anterior gonopod telopodite (at) curving laterad (Figure A14b, arrow); telopodital part (pt) of posterior gonopod laterally with serrate margin	*C. serratus*
