# Supplementary material for: Integrative Evidence Reveals Two New Species in the Millipede Genus Coxobolellus Pimvichai, Enghoff, Panha & Backeljau, 2020 and Reassigns Benoitolus siamensis (Attems, 1936) to the Genus Pseudospirobolellus Carl, 1912 (Diplopoda: Spirobolida: Pseudospirobolellidae)
Source: Animals (Basel). 2026 Jul 24;16(15):2304. doi: 10.3390/ani16152304 (PMC13463497; doi:10.3390/ani16152304)
Supplement: Supplementary file 1 [file animals-16-02304-s001.zip › Table S1.pdf]

Table S1. Specimens from which the COI and 16S rRNA gene fragments were sequenced. CUMZ, Museum of Zoology, Chulalongkorn University, Bangkok, Thailand; NHMD, Natural History Museum of Denmark; NHMW, Naturhistorisches Museum, Vienna, Austria; NHM, The Natural History Museum, London, United Kingdom. Names of countries are in capitals. Abbreviations after species names refer to the isolate of each sequence. GenBank accession numbers are indicated for each species; --- means no sequences were obtained.

	Voucher code	Locality	COI	16S rRNA
**Ingroup**				
**Genus *Apeuthes***				
A. maculatus Amc	NHMW-Inv. No.2395	South Annam, VIETNAM	MF187404	MF187360
A. maculatus Am26	NHMD-621697	Nha Trang, Bao Dai Villas Hotel, in garden, VIETNAM	MZ567159	MZ568653
A. fimbriatus BMP	CUMZ-D00144	Bach Ma Peak, Da Nang, VIETNAM	MZ567160	MZ568654
A. longeligulatus TPP	CUMZ-D00140	Tham Phet Po Thong, Klong Hard, Sa Kaeo, THAILAND	MZ567161	MZ568655
A. pollex SMR	CUMZ-D00141	Sra Morakot, Klongthom, Krabi, THAILAND	MZ567162	MZ568656
A. pollex SML	CUMZ-D00142	Koh 8, Similan islands, Phang-Nga, THAILAND	MZ567163	MZ568657
A. pollex WTS	CUMZ-D00143	Tham Sue Temple, Muang, Krabi, THAILAND	MZ567164	MZ568658
?*A. spininavis* ABB	CUMZ-D00145	Air Banun, Perak, MALAYSIA	MZ567165	MZ568659
**Genus *Atopochetus***				
A. anaticeps SVL	CUMZ- D00091	Srivilai temple, Chalermprakiet, Saraburi, THAILAND	MF187405	----
A. dollfusii DOL	NHM	Cochinchina, VIETNAM	MF187412	MF187367
A. helix SPT	CUMZ- D00094	Suan Pa Thong Pha Phum, Kanchanaburi, THAILAND	MF187416	MF187371
A. moulmeinensis TAK	CUMZ- D00095	km 87, Tha Song Yang, Tak, THAILAND	MF187417	MF187372
A. setiferus HPT	CUMZ- D00097	Hub Pa Tard, Lan-Sak, Uthaithani, THAILAND	MF187419	MF187374
A. spinimargo Ton27	NHMD- 00047013	Koh Yo, Songkhla, THAILAND	MF187423	MF187377
A. truncatus SML	CUMZ- D00101	Koh 8, Similan islands, Phang-Nga, THAILAND	MF187424	MF187378
A. uncinatus KMR	CUMZ- D00102	Khao Mar Rong, Bangsapan, Prachuapkhirikhan, THAILAND	MF187425	MF187379
A. weseneri Tos29	NHMD-00047003	Supar Royal Beach Hotel, Khanom, Nakhonsrithammarat, THAILAND	MF187431	MF187384
**Genus *Aulacobolus***				
A. uncopygus Auc	NHMW-Inv. No.2375	Nilgiris, South India, INDIA	MF187433	MF187386
**Genus *Benoitolus***				
B. birgitae BBG	NHMD 621687	Chiang Dao, Chiang Mai, THAILAND	MT328992	----

**Genus *Coxobolellus***				
C. albiceps Stpw	CUMZ-D00121	Tham Pha Tub, Muang District, Nan Province, THAILAND (green individual)	MT328994	MT328211
C. albiceps Stpl	CUMZ-D00122	Tham Pha Tub, Muang District, Nan Province, THAILAND (small, brown individual)	MT328993	----
C. albiceps TPB	CUMZ-D00123	Wat Tham Bampen Bun, Pan District, Chiang-Rai Province, THAILAND	MT328996	MT328213
C. albiceps Stvd	CUMZ-D00124	Tham Wang Daeng, Noen Maprang District, Phitsanulok Province, THAILAND	MT328995	MT328212
C. compactogonus SKR	CUMZ-D00134	Sakaerat Environmental Research Station, Wang Nam Khiao District, Nakhon Ratchasima Province, THAILAND	MT328998	MT328215
C. compactogonus KLC	CUMZ-D00135	Khao Look Chang, Pak Chong District, Nakhon Ratchasima Province, THAILAND	MT328997	MT328214
C. fuscus HKK	CUMZ-D00133	Kroeng Krawia waterfall, Sangkhla Buri District, Kanchanaburi Province, THAILAND	MT328999	MT328216
C. nodosus SPW	CUMZ-D00126	Chao Por Phawo Shrine, Mae Sot District, Tak Province, THAILAND	MT329000	MT328217
C. saratani PPLL2	CUMZ-D00153	Phu Pha Lom, Muang District, Loei Province, THAILAND	OP580097	OP580512
C. saratani PPLL	CUMZ-D00153-1	Phu Pha Lom, Muang District, Loei Province, THAILAND	OP580098	OP580513
C. serratoligulatus TCU	CUMZ-D00154	Tham Chan, Thong Saen Khan District, Uttaradit Province, THAILAND	OP580099	OP580514
C. serratoligulatus TCU2	CUMZ-D00154-1	Tham Chan, Thong Saen Khan District, Uttaradit Province, THAILAND	OP580100	OP580515
C. serratus KKL	CUMZ-D00132	Khao Kalok, Pran Buri District, Prachuap Khiri Khan Province, THAILAND	MT329001	MT328218
C. simplex TNP	CUMZ-D00136	Tham Pha Pha Ngam, Mae Prik District, Lampang Province, THAILAND	MT329002	----
C. tenebris KWP	CUMZ-D00119	Wat Khao Wong Phrohm-majan, Ban Rai District, Uthai Thani Province, THAILAND	MT329003	MT328219
C. tenebris TPL	CUMZ-D00120	Wat Tham Phrom Lok Khao Yai, Sai Yok District, Kanchanaburi Province, THAILAND	MT329004	MT328220
C. tigris TKP	CUMZ-D00130	Wat Tham Khao Plu, Pathio District, Chumphon Province, THAILAND	MT329005	MT328221
C. tigris TYE	CUMZ-D00131	Tham Yai I, Pathio District, Chumphon Province, THAILAND	MT329006	MT328222

C. transversalis Stpg	CUMZ-D00125	Tham Pha Tub, Muang District, Nan Province, THAILAND	MT329007	MT328223
C. valvatus TCD	CUMZ-D00127	Wat Tham Chiang Dao, Chiang Dao District, Chiang-Mai Province, THAILAND	MT329009	----
C. valvatus BRC	CUMZ-D00128	Tham Borichinda, Chom Thong District, Chiang-Mai Province, THAILAND	MT329008	MT328224
C. valvatus TST	CUMZ-D00129	Tham Sam Ta, Muang District, Mae Hong Son Province, THAILAND	MT329010	MT328225
C. sirindhornae Wptw1	CUMZ-D00156	Wat Pa Tham Wua, Pangmapa District, Mae Hong Son Province, THAILAND	PV612839	----
C. sirindhornae Wptw2	CUMZ-D00156-1	Wat Pa Tham Wua, Pangmapa District, Mae Hong Son Province, THAILAND	PV612840	----
C. kaengkrachanensis sp. nov. KKCP1	CUMZ-D00158	Kaeng Krachan, Kaeng Krachan District, Phetchaburi Province, THAILAND	PZ310494	PZ311613
C. kaengkrachanensis sp. nov. KKCP2	CUMZ-D00158-1	Kaeng Krachan, Kaeng Krachan District, Phetchaburi Province, THAILAND	PZ310495	PZ311614
C. planiprocessus sp. nov. NHYP1	CUMZ-D00159	Huai Yang waterfall, Thap Sakae District, Prachuap Khiri Khan Province, THAILAND	PZ310496	PZ311615
C. planiprocessus sp. nov. NHYP2	CUMZ-D00159-1	Huai Yang waterfall, Thap Sakae District, Prachuap Khiri Khan Province, THAILAND	PZ310497	PZ311616
**Genus *Leptogoniulus***				
L. sorornus BTN	CUMZ- D00109	Botanical Garden, Penang, MALAYSIA	MF187434	MF187387
**Genus *Litostrophus***				
L. chamaeleon PPT	CUMZ- D00111	Phu Pha terb, Mukdahan, THAILAND	MF187436	MF187389
L. saraburensis PKS	CUMZ- D00113	Phukhae Botanical Garden, Saraburi, THAILAND	MF187438	MF187391
L. segregatus Ls19	NHMD 621686	Koh Kut, Trad, THAILAND	MF187440	MF187394
**Genus *Macrurobolus***				
M. macrurus INT	CUMZ- D00147	Wat Tham Inthanin, Mae Sot District, Tak Province, THAILAND	MZ905519	----
**Genus *Madabolus***				
M. maximus Mm4	NHMD-00047007	de Toliara Province, Parc National de Bermaraha, South Bank of Manambolo River, Near Tombeau Vazimba, MADAGASCAR	MF187441	MF187395
**Genus *Narceus***				
N. annularis		No locality data	NC_003343.1	----

**Genus *Parabolus***				
P. dimorphus Pd34	NHMD-00047004	Dar es Salaam, TANZANIA	MF187442	MF187396
**Genus *Paraspirobolus***				
P. lucifugus		No locality data	AB608779.1	----
**Genus *Pelmatojulus***				
P. tigrinus Pt2	NHMD-00047008	Southern part of the Comoé N.P., 30 km north of Kakpin, CÔTE d'IVOIRE	MF187443	MF187397
P. togoensis Pto6	NHMD-00047006	Biakpa, GHANA	MF187444	MF187398
**Genus *Pseudospirobolellus***				
Pseudospirobolellus avernus GPG	CUMZ-D00117	Gua Pulai, Gua Musang, Kelantan, MALAYSIA	MT329011	MT328226
Pseudospirobolellus siamensis comb. nov. KCS	CUMZ-D00118	Koh Chuang, Sattahip, Chonburi, THAILAND	MT329012	MT328227
Pseudospirobolellus sp. KSM	CUMZ-D00161	Koh Sa-Med, Muang, Ra-Yong, THAILAND	PZ385172	PZ385173
**Genus *Rhinocricus***				
R. parcus Rp49	NHMD-00047009	Puerto Rico, USA	MF187449	MF187403
**Genus *Siliquobolellus***				
S. amicusdraconis HPT2	CUMZ- D00149	Hub Pa Tard, Lan-Sak, Uthaithani, THAILAND	OP174621	----
S. constrictus BYC	CUMZ- D00150	Ban Yang Chum, Kui Buri, Prachuap Khiri Khan, THAILAND	OP174622	----
S. prasankokae TPTG	CUMZ- D00148	Pha Thai, Ngao, Lampang, THAILAND	OP174623	----
**Genus *Trachelomegalus***				
T. sp. Tr54	ZMUC-00047012	Borneo Sabah, MALAYSIA	MF187445	----
T. cf. *hoplurus* Th	ZMUC-00047002	Borneo Sarawak, Niah, MALAYSIA	---	MF187399
**Genus *Trigoniulus***				
T. corallinus Tco15	NHMD-00047010	Vientiane, LAOS	MF187446	MF187400
**Outgroup**				
**Genus *Anurostreptus***				

A. barthelemyae Tlb	CUMZ-D00003	Thale-Ban N.P., Khuan-Don, Satun, THAILAND	KC519469	KC519543
**Genus *Thyropygus***				
T. allevatus Bb	CUMZ-D00013	BangBan, Ayutthaya, THAILAND	KC519479	KC519552
